# Stabilization of p18 by deubiquitylase CYLD is pivotal for cell cycle progression and viral replication

**DOI:** 10.1038/s41698-021-00153-8

**Published:** 2021-03-02

**Authors:** Yueshuo Li, Feng Shi, Jianmin Hu, Longlong Xie, Lin Zhao, Min Tang, Xiangjian Luo, Mao Ye, Hui Zheng, Min Zhou, Na Liu, Ann M. Bode, Jia Fan, Jian Zhou, Qiang Gao, Shuangjian Qiu, Weizhong Wu, Xin Zhang, Weihua Liao, Ya Cao

**Affiliations:** 1 Key Laboratory of Carcinogenesis and Cancer Invasion, Chinese Ministry of Education, Department of Radiology, Xiangya Hospital, Central South University, Changsha, China; 2 Cancer Research Institute and School of Basic Medical Science, Xiangya School of Medicine, Central South University, Changsha, China; 3 Key Laboratory of Carcinogenesis, Chinese Ministry of Health, Changsha, China; 4 Molecular Science and Biomedicine Laboratory, State Key Laboratory of Chemo/ Biosensing and Chemometrics, College of Biology, Hunan University, Changsha, China; 5 Institutes of Biology and Medical Sciences, Soochow University, Suzhou, China; 6 The Hormel Institute, University of Minnesota, Austin, MN USA; 7 Key Laboratory for Carcinogenesis and Cancer Invasion, Chinese Ministry of Education, Zhongshan Hospital, Shanghai Medical School, Fudan University, Shanghai, China; 8 Department of Otolaryngology Head and Neck Surgery, Xiangya Hospital, Central South University, Changsha, China; 9 Department of Radiology, Xiangya Hospital, Central South University, Changsha, China; 10 Molecular Imaging Research Center of Central South University, Changsha, Hunan China; 11 Research Center for Technologies of Nucleic Acid-Based Diagnostics and Therapeutics Hunan Province, Changsha, China; 12 National Joint Engineering Research Center for Genetic Diagnostics of Infectious Diseases and Cancer, Changsha, China

**Keywords:** Head and neck cancer, Ubiquitylation, Oncogenesis

## Abstract

p18 is a key negative regulator of cell cycle progression and mediates cell cycle arrest at the G1/S phase. Ubiquitination is the prime mechanism in regulating p18 protein abundance. However, so far no post- translational regulator, especially DUBs, has been identified to regulate the protein stability of p18. In this paper, we identified CYLD as a deubiquitinase of p18, which binds to and removes the K48-linked polyubiquitylation chains conjugated onto p18, thus stabilizing the p18 protein. Loss of CYLD causes the degradation of p18 and induces the G1/S transition. Epstein–Barr virus (EBV), is the human oncovirus etiologically linked to nasopharyngeal carcinoma (NPC). Here we found that EBV drives a replication passive environment by deregulating the CYLD-p18 axis. Functionally, CYLD inhibits cell proliferation and tumorigenesis through p18 in vivo. Restoring CYLD prevents EBV induced viral replication and tumor growth. Collectively, our results identify CYLD directly stabilizes p18 to regulate the cellular G1/S transition. The reconstitution of CYLD-p18 axis could be a promising approach for EBV-positive cancer therapy.

## Introduction

Cyclin-dependent kinase inhibitors (CKIs) are key negative regulators in cell cycle progression, they bind to cyclin-dependent kinases (CDKs) and block their association with cyclins^1,2^. Two CKI families are known, including the INK4 family (p16INK4A, p15INK4B, p18INK4C, and p19INK4D) and the CIP/KIP family (p21CIP1, p27KIP1 and p57KIP2)^3,4^. CKIs from the INK4 family interact with CDK4 or CDK6, and prevent the activation of the cyclin D/CDK4/6 kinases, resulting in decreased Rb phosphorylation, thereby inhibiting E2F transcription factors to activate the transcription of a plethora of genes involved in cell cycle progression from G1 into S phase^1,5^. The *p18* gene is a strong tumor suppressor in the INK4 family and downregulation of p18 leads to aberrant cell growth in glioblastoma multiforme (GBM), acute lymphoblastic leukemia (ALL), among others^6–8^.

Post translation modification mechanisms involving the ubiquitin-proteasome pathway mechanisms play a dominant role in regulating protein abundance^9,10^. Removal of the ubiquitin signal catalysed by deubiquitylase enzymes (DUBs) contributes to the regulation of diverse cellular processes^11^. Deubiquitylase was found to play an important role in multiple cellular functions and imbalances in deubiquitylase is associated with multiple diseases, including cancer, inflammation, and infection^12,13^. So far, about 100 deubiquitylases belonging to six families have been identified^14^. The cylindromatosis (CYLD) protein is a deubiquitylase in the ubiquitin-specific processing protease (USP) family^15^. As a tumor suppressor, *CYLD* was identified as mutated in familial cylindromatosis and loss of CYLD has also been implicated in several other malignancies, such as colon and hepatocellular carcinomas, multiple myeloma, melanoma, and breast cancer^16,17^. The CYLD protein has three cytoskeleton-associated proteinglycine-rich (CAP-Gly) domain, two proline-rich (PR) domain, and a USP domain, which primary removes K63 or M1 polyubiquitin chains from target proteins^18^. CYLD controls cell survival, inflammation, proliferation, and tumorigenesis by regulating its targets in multiple signaling pathways^19–23^.

Epstein-Barr virus (EBV) infection is ubiquitous and associated with the development of a variety of diseases but especially cancers, including nasopharyngeal carcinoma (NPC), gastric cancer, and Burkitt’s lymphoma (BL)^24,25^. The EBV life cycle comprises two stages, latent infection and lytic replication. During latent infection, EBV expresses a limited number of viral genes and EBV episomes replicate only once in S phase^26^. In the lytic stage, transcriptional activator Zta(BZLF1), together with EBV replication proteins like EAD(BMRF1) and BALF2/5 induce intense viral DNA replication and over 80 viral proteins are expressed in this stage^27^. This reactivation corresponds with the emergence of human cancers and antibodies targeting early lytic proteins, including BZLF1, BMRF1, and BALF2, as well as EBV DNA load testing, have been used in diagnosing NPC at an early stage as well as locally recurrent disease^28–30^. Viral genome replication appears to be the core event in lytic infection^31^. EBV encodes BPLF1 and promotes cell entry into S phase deregulation of the cell cycle^32^. Besides EBV replication proteins, cellular factors are also important in supporting environmental advantages for viral lytic replication.

In this research project, we identified CYLD as a deubiquitinase that directly cleaves polyubiquitination chains of p18 and stabilizes its protein level, thereby negatively regulating cell cycle G1-S progression. We also found that EBV deregulates the CYLD-p18 axis, which contributes to viral DNA replication and tumor growth. Our findings provide new insights into the ubiquitination mechanism of mediation of CKI p18 by CYLD and EBV deregulation of the host cell cycle machinery to promote replication of viral DNA.

## Results

### EBV inhibits CYLD expression and contributes to tumorigenesis in NPC

**Fig. 1:**
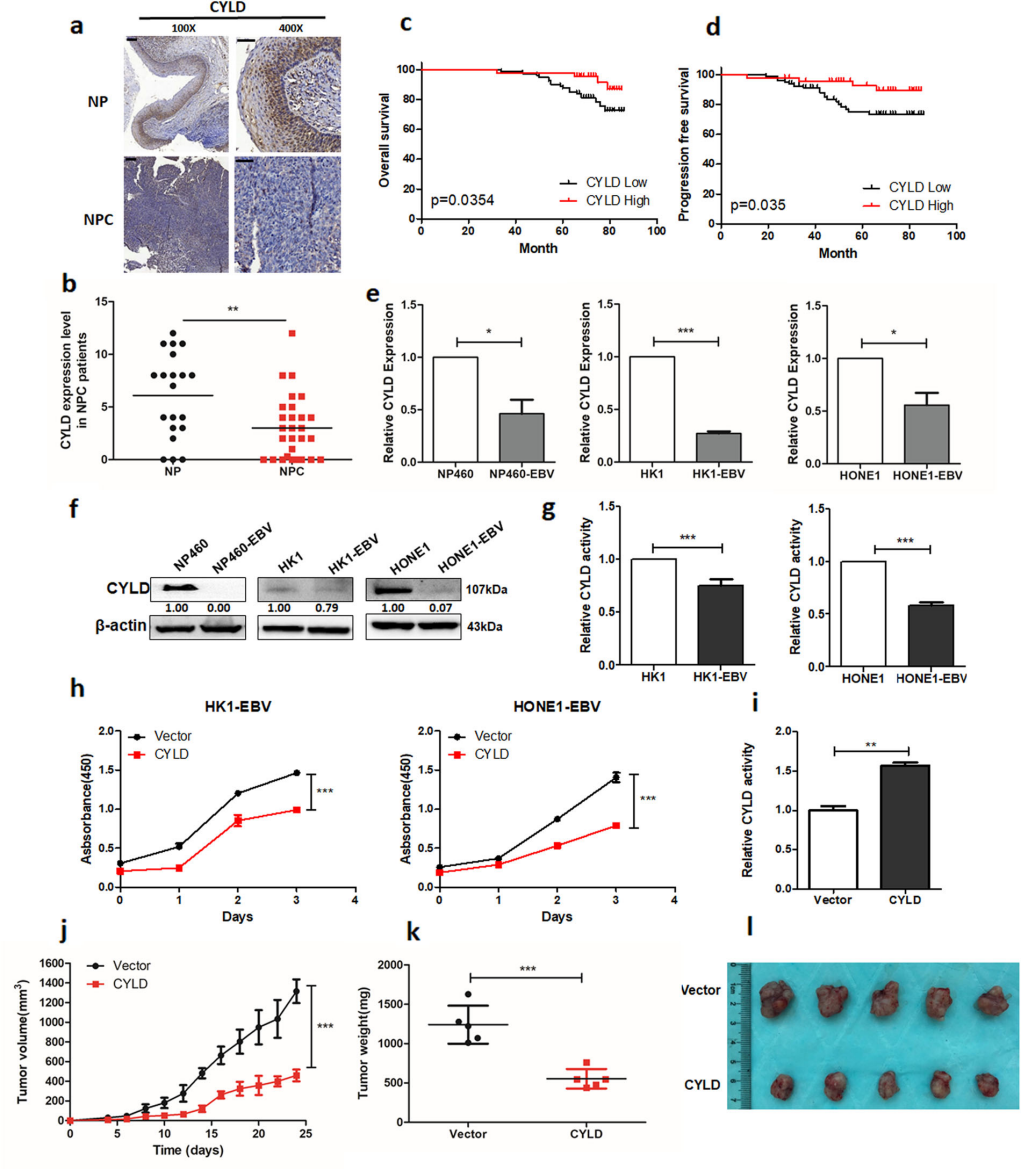
EBV inhibits CYLD expression and contributes to tumorigenesis in NPC. **a** Representative IHC staining of CYLD expression from pathological sections of NPC patients (100×: scale bar, 100 μm; 400×: scale bar, 50 μm). **b** Histoscores of CYLD expression in NPC patients (*n* = 29) compared to nasopharyngeal (NP) tissue (*n* = 20). **c** Overall survival and **d** Progression free survival rates of NPC patients with low (*n* = 71) or high (*n* = 58) expression levels of CYLD were estimated by the Kaplan–Meier method using log-rank test. Group according to CYLD median expression. **e** mRNA and **f** protein expression of CYLD in NP460/NP460-EBV, HK1/HK1-EBV, and HONE1/HONE1-EBV cell lines. **g** DUB activity measurement of CYLD in HK1/HK1-EBV, HONE1/HONE1-EBV cells. **h** HK1-EBV and HONE1-EBV cells were infected with a lentivirus and proliferation was monitored using CCK8 assays at the indicated time points. Statistical significance was determined by a two-tailed, unpaired Student’s t test. The indicated cells (5 × 10^6^) were subcutaneously injected into mice. **i** CYLD activity in tumor extracts. **j** Tumor growth, **k** tumor weight, and **l** tumor images are shown. (Data are represented as mean ± SEM (*n* = 3). Differences were considered significant at *p < 0.05, **p < 0.001, ***p < 0.001).

Kaplan-Meier curves showed that patients with head and neck squamous cell cancer (HNSCC) with low expression of CYLD had a shorter overall survival (*n* = 283; median survival time: 26.8 months vs. 65.7 months; Supplementary Fig. 1a). NPC is an important subtype of HNSCC. We analyzed CYLD level in the Oncomine database, and found that the mRNA level of *CYLD* is significantly down-regulated in NPC tissues (Supplementary Fig. 1b). Lower protein levels of CYLD were also detected in NPC tissues compared to nasopharyngeal tissues both in pathological sections and tissue microarray of NPC patients (Fig. 1a, b, Supplementary Fig. 1c, d). Then two NPC tumor tissue microarrays were used to investigate the prognostic role of CYLD. Based on the median score of CYLD, 129 NPC patients were divided into two groups. The patients with low levels of CYLD (*n* = 71) had a shorter overall survival and progression free survival compared to those with higher CYLD (*n* = 58) levels, and there is higher recurrence rates in patients with low CYLD expression (Fig. 1c, d). Another tissue microarray got the similar results (Supplementary Fig. 1e). All of the tumor biopsies were obtained before treatment and patients’ clinical characteristics are listed in Supplementary Tables 1, 2 and 3.

EBV infection is known to play a crucial role in NPC tumorigenesis. Thus we evaluated whether EBV is involved in CYLD down-regulation. The expression of CYLD in EBV-uninfected and EBV-infected cells was examined and results showed that mRNA and protein levels of CYLD were significantly reduced in the EBV- infected immortalized nasopharyngeal epithelial cell lines, NP460hTERT-EBV, and also the nasopharyngeal carcinoma cell line, HK1-EBV and HONE1-EBV (Fig. 1e, f). Further, analysis of DUB activity showed that CYLD levels led to decreased enzyme activity (Fig. 1g, Supplementary Fig. 2). Accelerated cellular proliferation is a general characteristic in EBV infected cells, so is true in our cell model (Supplementary Fig. 3). To investigate the effect of CYLD on EBV-mediated tumor cell growth, we conducted cell proliferation assays with CCK8. Results showed that overexpression of CYLD in HK1-EBV and HONE1-EBV cells inhibits cell proliferation (Fig. 1h). Further, we performed a xenograft tumor formation assay. HONE1-EBV cells stably transfected with CYLD or empty vector were injected subcutaneously into nude mice. Compared with empty vector infected cells, the mice injected with CYLD expressing HONE1-EBV cells showed increased CYLD activity and decreased tumor growth throughout the experiment (Fig. 1i, j). Overall, the average volume and weight of xenograft tumors in mice injected with HONE1-EBV-CYLD cells were significantly lower compared to tumor in mice injected with vector cells (Fig. 1k, l).

### EBV accelerates cell G1/S transition through CYLD inhibition

**Fig. 2:**
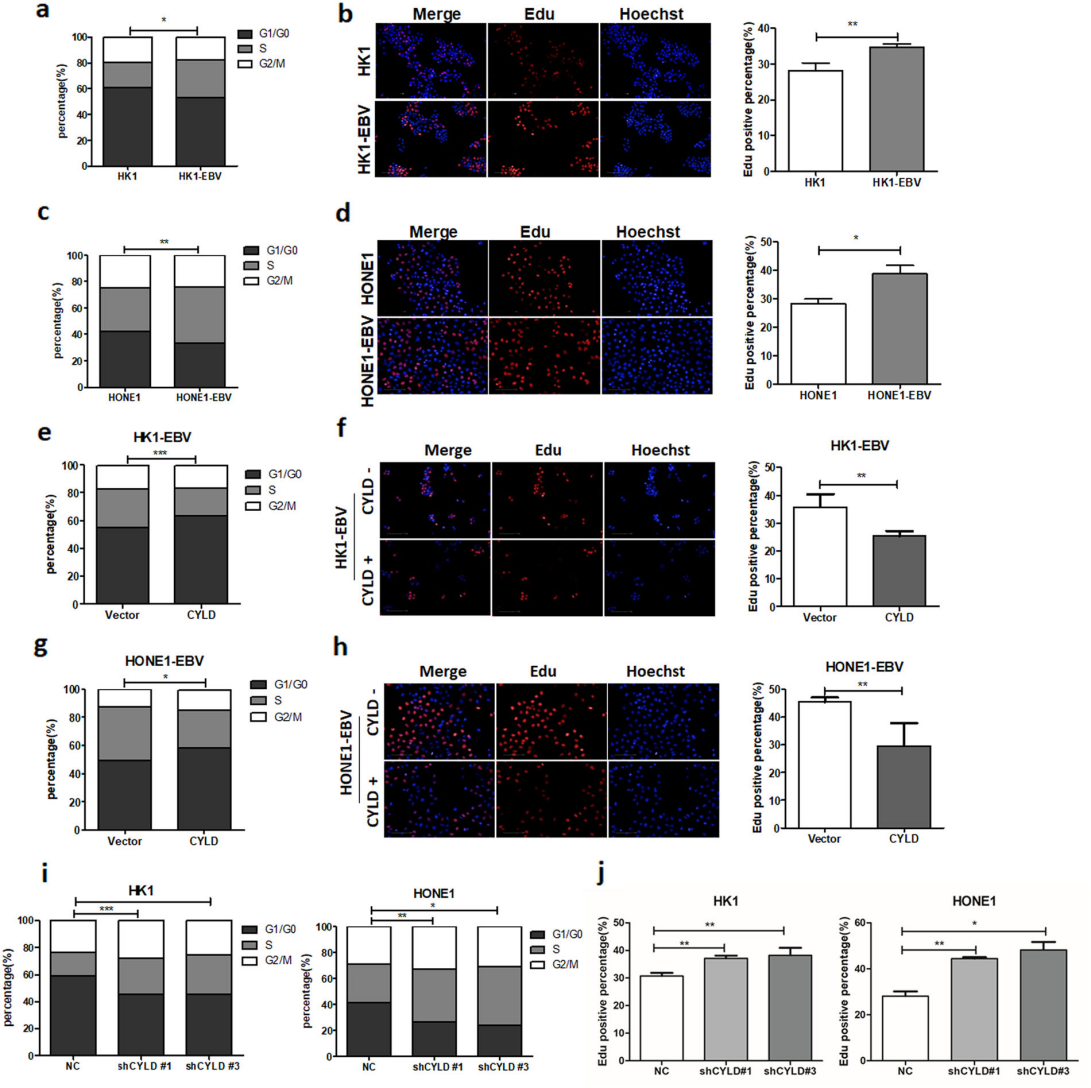
EBV accelerates cell G1/S transition through CYLD inhibition. **a**, **c** HK1-EBV and HONE1-EBV cells was examined by staining with propidium iodide and analyzed by flow cytometry. **b**, **d** Edu staining analysis of S-phase cell cycle in HK1-EBV and HONE1-EBV cells. Average Edu intensity above 500 is considered positive. Nuclei were stained with Hoechst. (Scale bar=100 μm) **e, g** Overexpression of CYLD in HK1-EBV and HONE1-EBV cells was examined by staining with propidium iodide and analyzed by flow cytometry. **f, h** Edu staining analysis of S-phase cell cycle in control and CYLD overexpressing cells. Average Edu intensity above 500 is considered positive. Nuclei were stained with Hoechst. (Scale bar=100 μm). **i** HK1 and HONE1 cells infected with CYLD lentiviral shRNAs were stained with propidium iodide and analyzed by flow cytometry. **j** S-phase cell was analyzed by Edu staining. Nuclei were stained with Hoechst. (Scale bar=100 μm). (Data are represented as mean ± SEM (*n* = 3). Differences were considered significant at *p < 0.05, **p < 0.01, ***p < 0.001).

To further assess the function of CYLD in EBV-mediated tumor cell growth. Cell cycle distribution in HK1, HONE1 and EBV infected HK1-EBV, HONE1-EBV cells were analyzed by flow cytometry after serum starvation. The percentage of cells in S phase was increased in EBV infected HK1-EBV, HONE1-EBV cells (Fig. 2a, c, and Supplementary Fig. 4). And similar result was obtained from the Edu assay, the percentage of cells in S phase was increased in HK1-EBV and HONE1-EBV cells (Fig. 2b, d). Meanwhile we performed GSEA (Gene Set Enrichment Analysis) using 31 NPC data sets (GES12452) focusing on genes in cell signaling pathways with differential expression of CYLD. The low and high groups were split by median CYLD expression, and the curve was obtained by high groups /low groups. Which showed that CYLD was negatively associated with cell cycle (Supplementary Fig. 5a). Thus we examined the effects of CYLD expression on EBV (+) cell cycle distribution. Cell cycle analysis by flow cytometry was performed after serum starvation. The percentage of cells in S phase was decreased after overexpression of CYLD in HK1-EBV and HONE1-EBV cells (Fig. 2e, g and Supplementary Fig. 5b). And similar result was obtained from the Edu assay (Fig. 2f, h). To further confirm the role of CYLD, stable CYLD knockdown HK1 and HONE1 cells were constructed using a lentiviral-based CYLD small hairpin RNA. As predicted, CYLD knockdown increased the percentage of cells in S phase (Fig. 2i, j, Supplementary Fig. 5c, d). These results strongly suggest that the CYLD delays the G1/S transition, and EBV interferes with CYLD expression to accelerate host cell G1/S progression.

### CYLD regulates cell G1/S progression in a p18-dependent manner

**Fig. 3:**
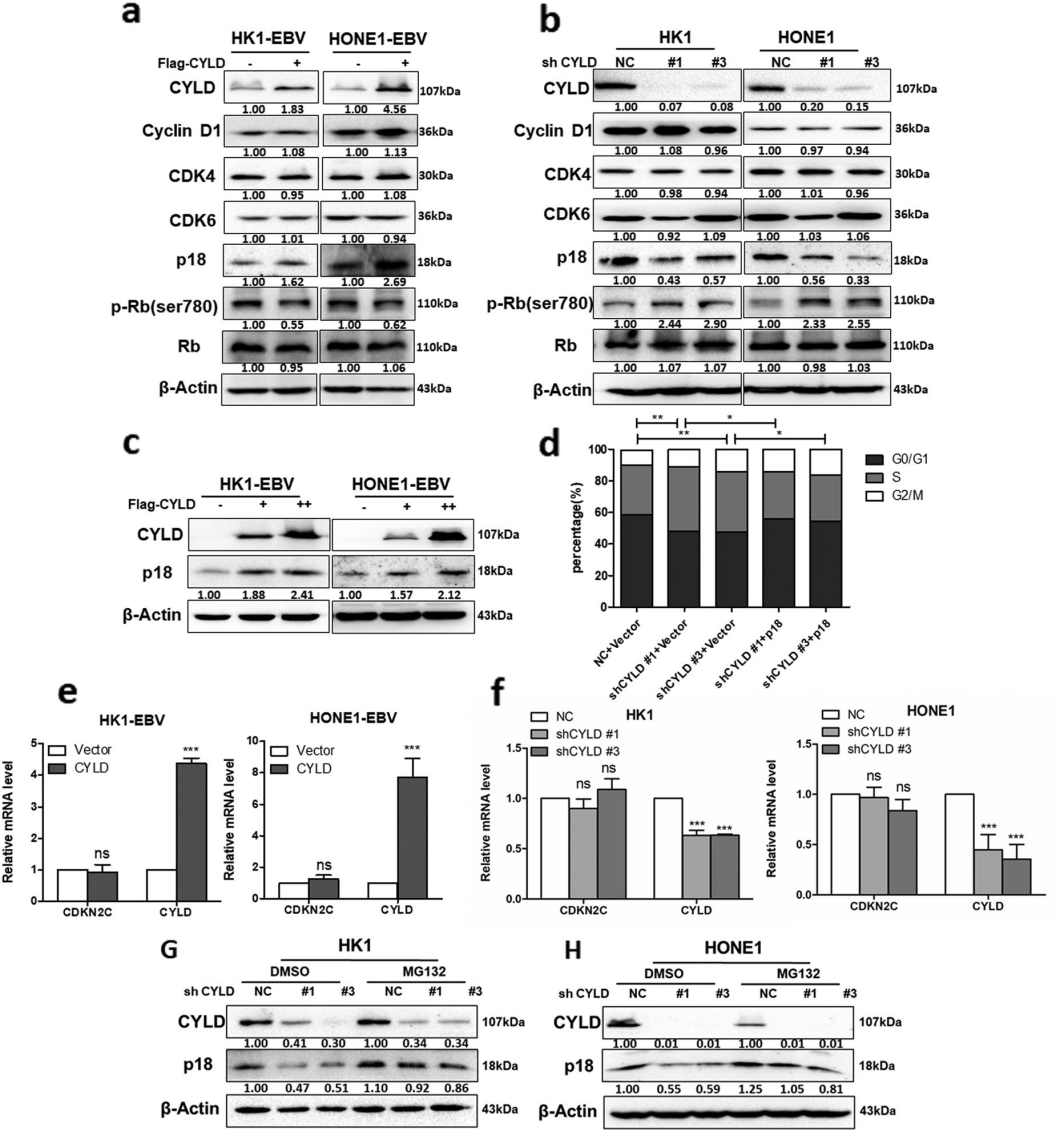
CYLD regulates the protein level of p18. **a** HK1-EBV and HONE1-EBV cells transfected with CYLD were immunoblotted with antibodies against the indicated proteins. **b** HK1 and HONE1 cells were infected with CYLD lentiviral shRNAs and cell lysates were immunoblotted with antibodies against the indicated proteins. **c** Increasing amounts of CYLD were transfected into HK1-EBV and HONE1-EBV cells, and total protein was extracted from these cells and subjected to Western blotting using anti-CYLD, anti-p18, or anti-β-actin. **d** HONE1 cells were infected with the indicated lentiviral shRNAs followed by transfection with the indicated constructs, and then stained with propidium iodide and analyzed by flow cytometry. Total RNA either from cells infected with **e** CYLD or from cells transfected with the indicated **f** lentiviral shRNAs was isolated and subjected to qPCR. The error bars represent the S.E.M. of triplicate measurements. **g** HK1 and **h** HONE1 cells infected with the indicated lentiviral shRNAs were treated with MG132 (20 μM) for 6 h and the indicated proteins were analyzed by Western blotting. (Data are represented as mean ± SEM (*n* = 3). Differences were considered significant at *p < 0.05, **p < 0.01, ***p < 0.001).

To identify targets of CYLD, we examined the protein level of cyclins, CDKs, and CKIs, as well as the phosphorylation of retinoblastoma protein (pRb). Results (Fig. 3a, b) indicated that neither overexpression or knockdown of CYLD changed the expression of cyclin D or CDK4 or 6, but influenced the protein level of p18 and pRb, and increasing CYLD expression caused an elevation in p18 levels in a dose-dependent manner in all cell lines (Fig. 3c). We also determined whether other CKIs, including the INK4 and CIP/KIP families, as well as p53 were affected, but no significant changes in protein level was observed in these CKIs and p53 (Supplementary Fig. 6a, b). We next determined whether p18 is required for the function of CYLD in the G1/S transition. The percentage of cells in S phase was determined by performing PI staining. Cell cycle analysis by flow cytometry was performed after serum starvation. Accelerated G1/S transition in CYLD knockdown cells was prevented with exogenous p18 (Fig. 3c, Supplementary Fig. 7). This result suggests that CYLD depends on p18 to regulate G1/S transition. Neither CYLD knockdown nor overexpression altered the mRNA level of *p18*, which indicates that CYLD may regulate p18 at the protein level (Fig. 3e, f). Further, treatment with the proteasome inhibitor MG132 indicated that CYLD regulates the protein levels of p18 by blocking its proteasomal degradation (Fig. 3g, h).

### CYLD regulates p18 by deubiquitination

**Fig. 4:**
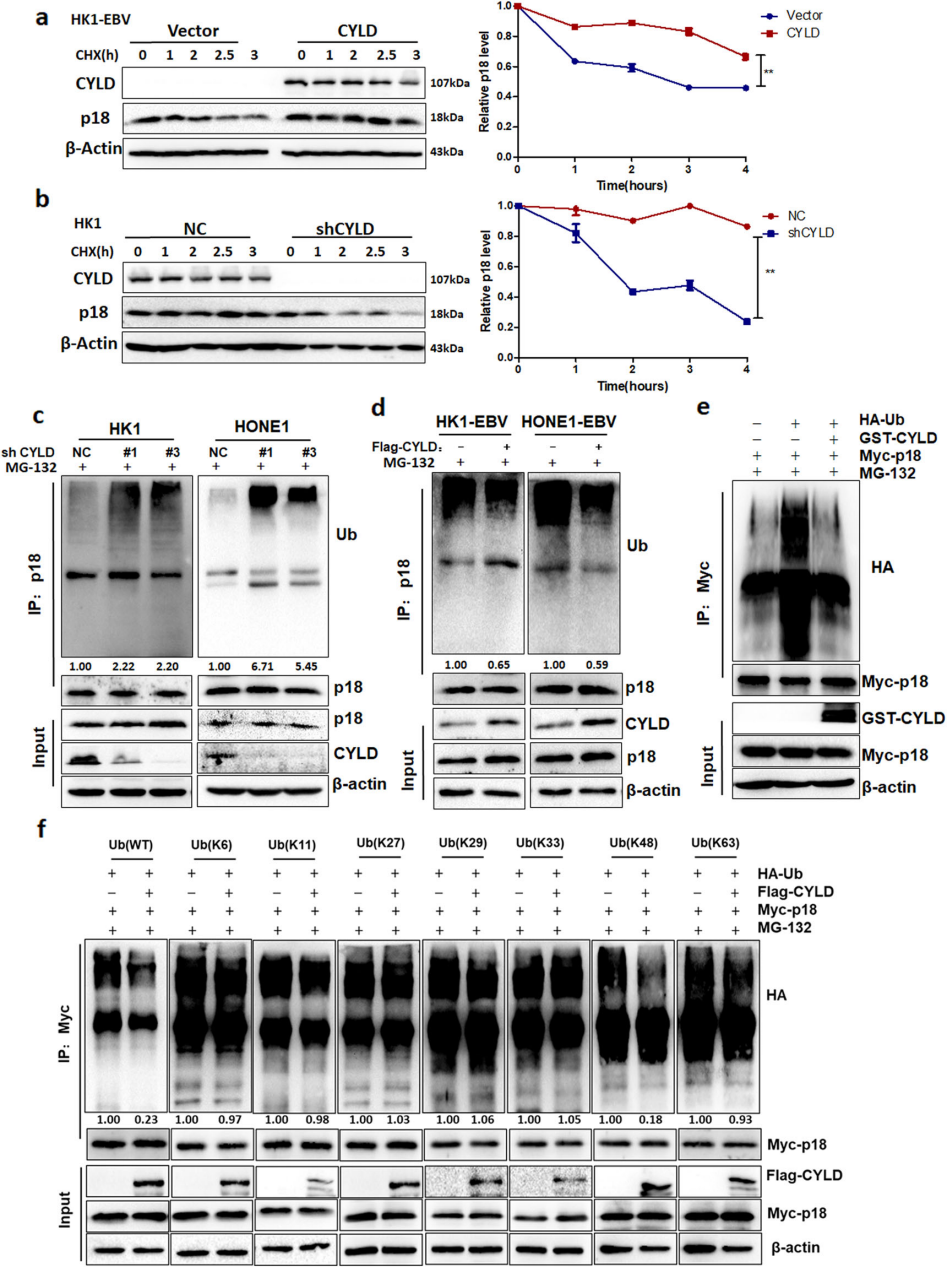
CYLD removes K48-linked poly-Ub in p18. **a** HK1-EBV cells transfected with the indicated constructs were treated with 20 μg·mL^−1^ CHX, collected at the indicated time points, and immunoblotted with anti- CYLD, anti-p18, or anti-β-actin. Quantification of the p18 levels relative to β-actin expression is shown (**p < 0.01). **b** HK1 cells transfected with the indicated lentiviral shRNAs were treated with 20 μg.mL^−1^ CHX, collected at the indicated time points, and immunoblotted with anti-CYLD, anti-p18, or anti-β-actin. Quantification of the p18 levels relative to β-actin expression is shown. **c** HK1-EBV and HONE1-EBV cells infected with CYLD were treated with MG132 (20 μM) for 6 h before harvest. The p18 protein was immunoprecipitated with a p18 antibody, and the immunoprecipitates were probed with anti-Ub or anti-p18. **d** HK1 and HONE1 cells infected with the indicated lentiviral shRNAs were treated with MG132 (20 μM) for 6 h before harvest. The p18 protein was immunoprecipitated with a p18 antibody, and the immunoprecipitates were probed with anti-Ub or anti-p18. **e** Ubiquitylated Myc-p18 was incubated with GST-tagged CYLD or not. After coincubation, Myc-p18 was immunoprecipitated using an anti-Myc antibody, and the immunoprecipitates were probed using antibodies against HA and Myc. Recombinant GST-tagged CYLD were analyzed using SDS/PAGE. **f** Myc-p18 and various HA-ubiquitin mutants were transfected into 293 T cells infected or not with Flag-CYLD. The cells were treated with a 20 μM concentration of the proteasome inhibitor MG132 for 6 h. Myc-p18 was immunoprecipitated with an Myc antibody, and the immunoprecipitates were probed with anti-HA and anti-Myc. (Data are represented as mean ± SEM (*n* = 3). Differences were considered significant at **p < 0.01).

Because CYLD contributes to the protein levels of p18, we further determined whether CYLD stabilizes p18. Cycloheximide (CHX) was used to inhibit protein biosynthesis, and then total protein extracts were collected at the indicated time points. Results showed that the half-life of the p18 protein is significantly prolonged by overexpression of CYLD (Fig. 4a, Supplementary Fig. 6c). In contrast, knockdown of CYLD substantially decreased the half-life of p18 (Fig. 4b, Supplementary Fig. 6d). Further, we purified CYLD and ubiquitylated p18, then incubated these two proteins in a cell-free system. The results showed that CYLD increase the protein level of p18 (Supplementary Fig. 6e).These results suggest that CYLD does indeed stabilizes p18. Furthermore, to understand the potential mechanism as to how CYLD regulates the stability of p18, the levels of polyubiquitylation of p18 were measured in NPC cells. Knockdown of CYLD significantly increased the polyubiquitylation of p18 (Fig. 4c), whereas, overexpression of CYLD reduced the levels of polyubiquitylation of p18 (Fig. 4d). To verify that p18 is a direct substrate of CYLD, we purified CYLD and ubiquitylated p18 and incubated these two proteins in a cell-free system. As expected, CYLD decreased p18 polyubiquitylation in vitro (Fig. 4e).These data indicate that CYLD directly deubiquitylates p18. To investigate which type of poly-Ub chain on p18 is cleaved by CYLD, we used 293T cells transfected with Myc-p18, Flag-CYLD, together with HA-tagged ubiquitin mutants (K6, K11, K27, K29, K33, K48, or K63). Overexpression of CYLD significantly decreased K48-linked poly-Ub, but not any other isopeptide-linked (K6, K11, K27, K29, K33, or K63) poly-Ub (Fig. 4f). This result suggests that CYLD removes K48-linked polyubiquitylation chains on p18.

### CYLD interacts with p18

**Fig. 5:**
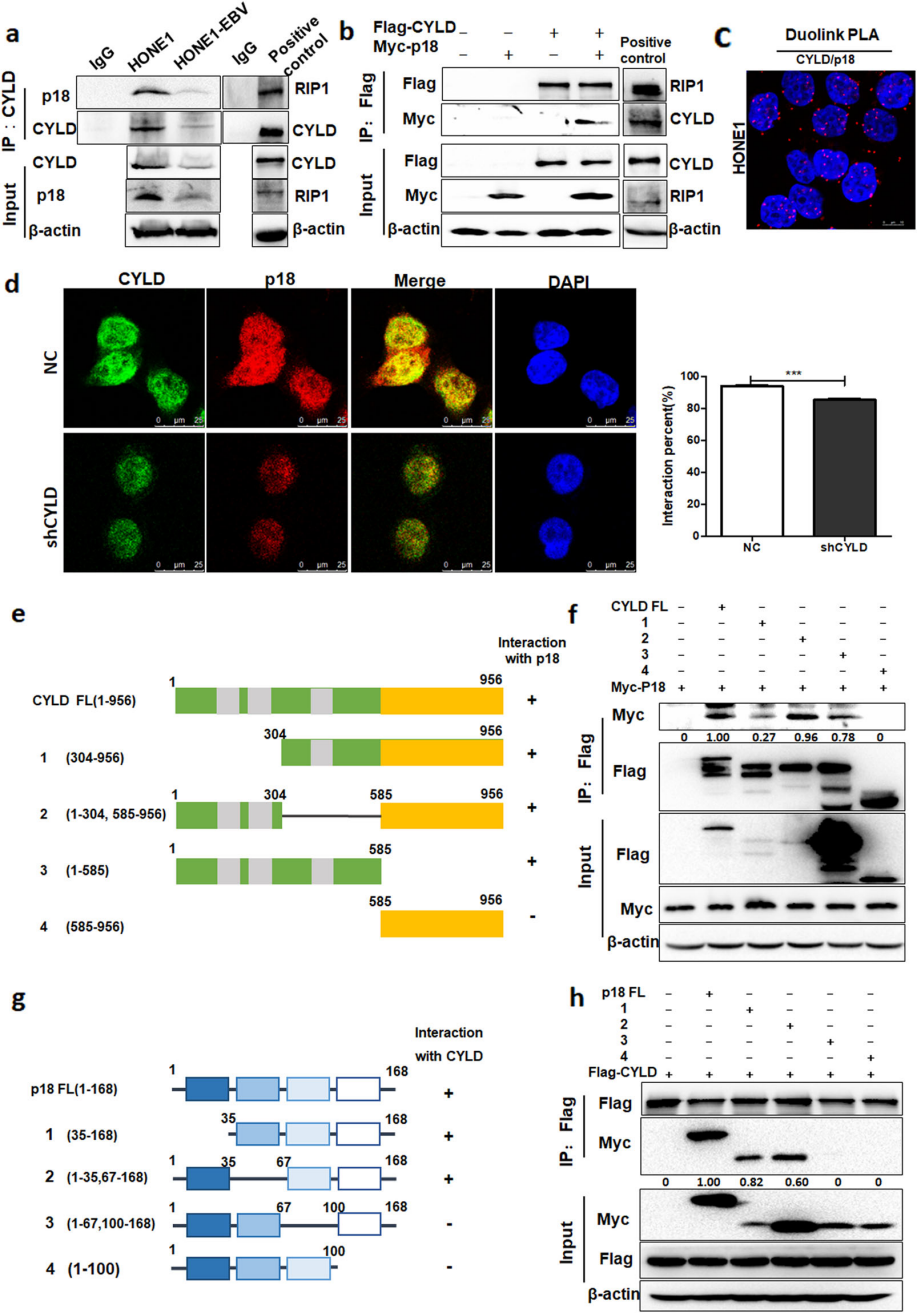
CYLD interacts with p18. **a** Immunoprecipitates were analyzed with CYLD antibodies in HONE1 and HONE1-EBV cells. The arrow points to the destination band. Immunoprecipitates of RIP1 was taken as positive control. **b** 293T cells were transfected with Flag-CYLD and Myc-p18, and he cell lysates were subjected to immunoprecipitation with Flag antibodies, then the immunoprecipitates were probed with anti-Flag or anti-p18. Immunoprecipitates of Flag-CYLD and Myc-RIP1 was taken as positive control. **c** Proximity ligation assay indicating the interaction of CYLD and p18 in HONE1 cells (red: PLA positive signal; blue: DAPI, scale bar = 10 μm). **d** Endogenous CYLD (green) and p18 (red) in HONE1-NC and -shCYLD cells was visualized by immunofluorescence with anti-CYLD and anti-p18. DNA was stained with DAPI, and a merged view of the red and green channels within the same field is shown (merge) (scale bar, 10 μm). The interaction percent was calculated by Image J. **e** Schematic representation of the Flag-tagged full-length CYLD (FL) and its various deletion mutants, including N-terminal 1-303 deletion (1), Middle domain deletion (2), N-terminal (3) and C-terminal (4) constructs. **f** 293 T cells transfected with the indicated constructs were disrupted. The cell lysates were subjected to immunoprecipitation with anti-Flag. **g** Schematic representation of the Myc-tagged full-length p18 (FL) and its various deletion mutants, including N-terminal 1-34 deletion (1), N-terminal 36-66 deletion (2), C-terminal 68-99 deletion (3) and C-terminal 101-168 deletion (4) constructs. **h** 293 T cells transfected with the indicated constructs were disrupted. The cell lysates were subjected to immunoprecipitation with anti-Flag, and the immunoprecipitates were then probed with anti-Myc and anti-Flag. (Data are represented as mean ± SEM (*n* = 3). Differences were considered significant at ***p < 0.001).

To better understand how CYLD regulates p18, we examined whether CYLD interacts with p18. CYLD was immunoprecipitated in HONE1 and HONE1-EBV cells. The p18 protein was detected in CYLD immunoprecipitated complexes and the protein–protein interaction with CYLD (Fig. 5a). The interaction of CYLD and RIP1 (Receptor Interaction protein 1) was taken as positive control. To further confirm the physiological interaction, Flag-CYLD and Myc-p18 were transfected into 293T cells, and co-immunoprecipitation was performed using a Flag antibody. The result showed that Myc-p18 was detected in the Flag-CYLD immunoprecipitates (Fig. 5b). The interaction of Flag-CYLD and Myc-RIP1 was taken as positive control. Immunofluorescent staining revealed that the co-localization of CYLD and p18 mainly occurred in the nucleus. Knockdown of CYLD leads to diminished CYLD and p18 fluorescence and consequent reduction in co-localization (Fig. 5d). Meanwhile, we used an in situ proximity ligation assay (PLA) to examine if a direct interaction occurred between CYLD and p18^33^. Positive fluorescent signals were observed, which indicates CYLD interacts directly with p18 (Fig. 5c). Additionally, to map the region of CYLD required for p18 binding, we constructed a series of CYLD-deletion mutants (Fig. 5e). The co-IP analysis revealed that the N-terminal region, especially amino acids 1–304 of CYLD, was critical for the interaction between CYLD and p18 (Fig. 5f). Immunofluorescent staining also convinced that N-domain of CYLD is responsible for the interaction to p18 (Supplementary Fig. 8). Conversely, the C terminus (amino acids 67-168) of p18 mediated the physical interaction with CYLD (Fig. 5g, h). Collectively, these results demonstrated that p18 is a bona fide interacting protein for CYLD.

### CYLD inhibits EBV lytic replication through p18

**Fig. 6:**
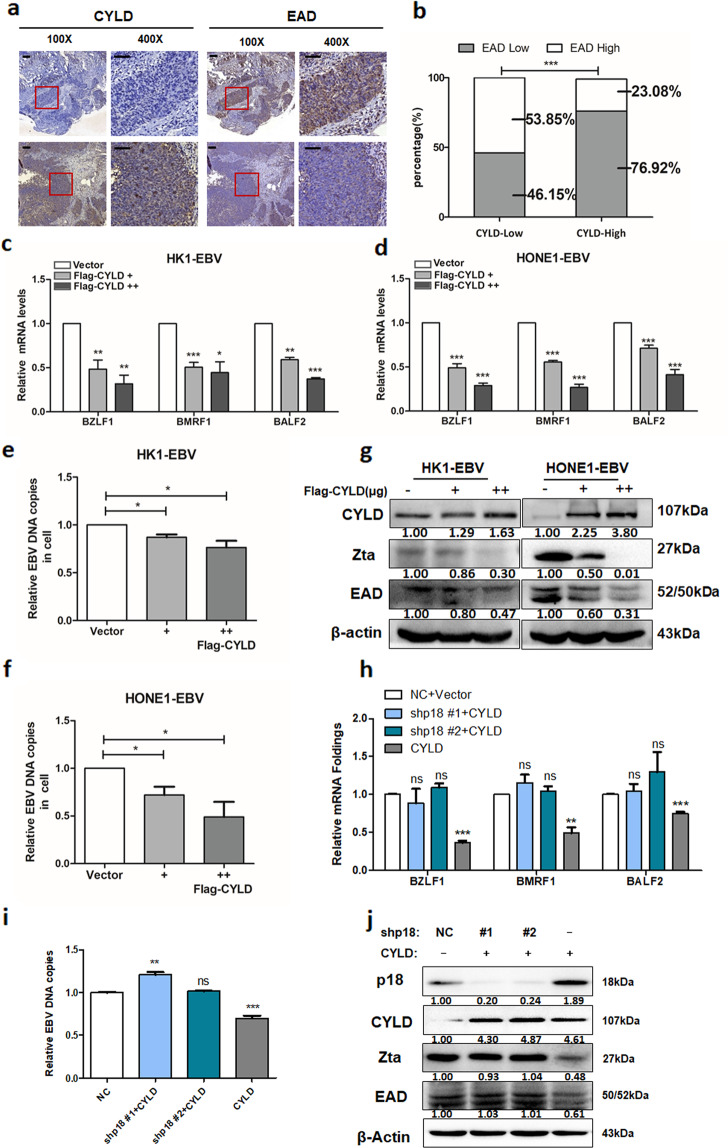
CYLD inhibits EBV reactivation dependent on p18. **a** Representative IHC staining of CYLD and EAD expression taken from pathological sections of NPC patients (100×: scale bar, 100 μm; 400×: scale bar, 50 μm). **b** EAD expression level was calculated based on CYLD expression. High and low groups were according to median expression. **c**, **d** HK1-EBV and HONE1-EBV cells infected with vector or CYLD and total RNA from cells was isolated and subjected to real-time PCR. **e**, **f** Real-time PCR showing EBV DNA copy levels of EBV in HK1-EBV and HONE1-EBV cells. HONE1-EBV cells were infected with p18 lentiviral shRNAs and then transfected with the indicated constructs. **g** Cell lysates were then extracted and subjected to Western blotting. **h** Total RNA and **i** gDNA were isolated and subjected to real-time PCR. **j** Cell lysates were immunoblotted with antibodies against the indicated proteins. β-Actin was used as a control to confirm equal loading of protein. (Data are represented as mean ± SEM (*n* = 3). Differences were considered significant at *p < 0.05, **p < 0.01, ***p < 0.001).

Viral replication relies on host cell cycle progression and EBV reactivated replication is closely associated with cancer. Therefore, the relationship of CYLD and EBV replication was analyzed in NPC tissues and a tumor tissue microarray. The expression of CYLD and EAD was detected by IHC and EAD represented EBV reactivation level (Fig. 6a, Supplementary Fig. 9a). The expression level of CYLD that was above or equal to the median score of 4 was regarded as the CYLD-high expressing group, whereas those below the median score were CYLD-low expressing group. In both NPC tissues and the tumor tissue microarray, increased EAD positivity was more frequently observed in CYLD-low expressing group than in CYLD-high expressing group (Fig. 6b, Supplementary Fig. 9b). A significant negative correlation calculated by the Pearson χ2 test was observed between the two proteins (NPC patients: *r* = −0.46; NPC microarray: *r* = −0.40. *p* < 0.001, Supplementary Tables 4 and 5). To investigate the effect of the CYLD-p18 axis in EBV lytic replication, CYLD was transfected into HK1-EBV and HONE1-EBV cells. We then further evaluated EBV lytic replication by the expression of immediate early gene *BZLF1 (Zta)*, early gene *BMRF1 (EAD)*, and *BALF2* in the EBV lytic phase, as well as EBV DNA loads. Results showed that the mRNA level of *BZLF1, BMRF1*, and *BALF2* and the protein expression of Zta and EAD were reduced in the CYLD overexpressing group (Fig. 6c, d, g and Supplementary Fig. 9c). Detection of EBV DNA loads in cells and culture supernatant fractions showed that EBV DNA loads were reduced by transfection of CYLD (Fig. 6e, f and Supplementary Fig. 9d), which indicated the decrease of EBV DNA replication and leading to the reduction of viral DNA release to extracellular. In addition, the expression of CYLD, Zta, and EAD in xenograft tumors was examined by Western blot and results showed that the EBV lytic replication markers Zta and EAD were depressed by CYLD overexpression (Supplementary Fig. 9e). Results of Real-time Quantitative PCR analysis in the detection of EBV DNA copies in xenograft tumors also confirmed the effect of CYLD on EBV replication (Supplementary Fig. 9f). These results indicate that CYLD inhibits EBV reactivation and then affects EBV DNA replication.

To further investigate whether the inhibition of EBV replication caused by CYLD is p18 dependent, stable p18 knockdown HONE1-EBV cells were constructed with a lentiviral-based p18 small hairpin RNA. Overexpression of CYLD in p18 knockdown cells had no effect on the expression of immediate early gene *BZLF1* (ZTA), early gene *BMRF1* (EAD), or *BALF2* in EBV lytic phase, as well as EBV DNA loads (Fig. 6h–j). These results strongly suggest that the CYLD regulates EBV replication in a p18-dependent manner.

### CYLD exerts a p18-dependent tumor-suppressing function

**Fig. 7:**
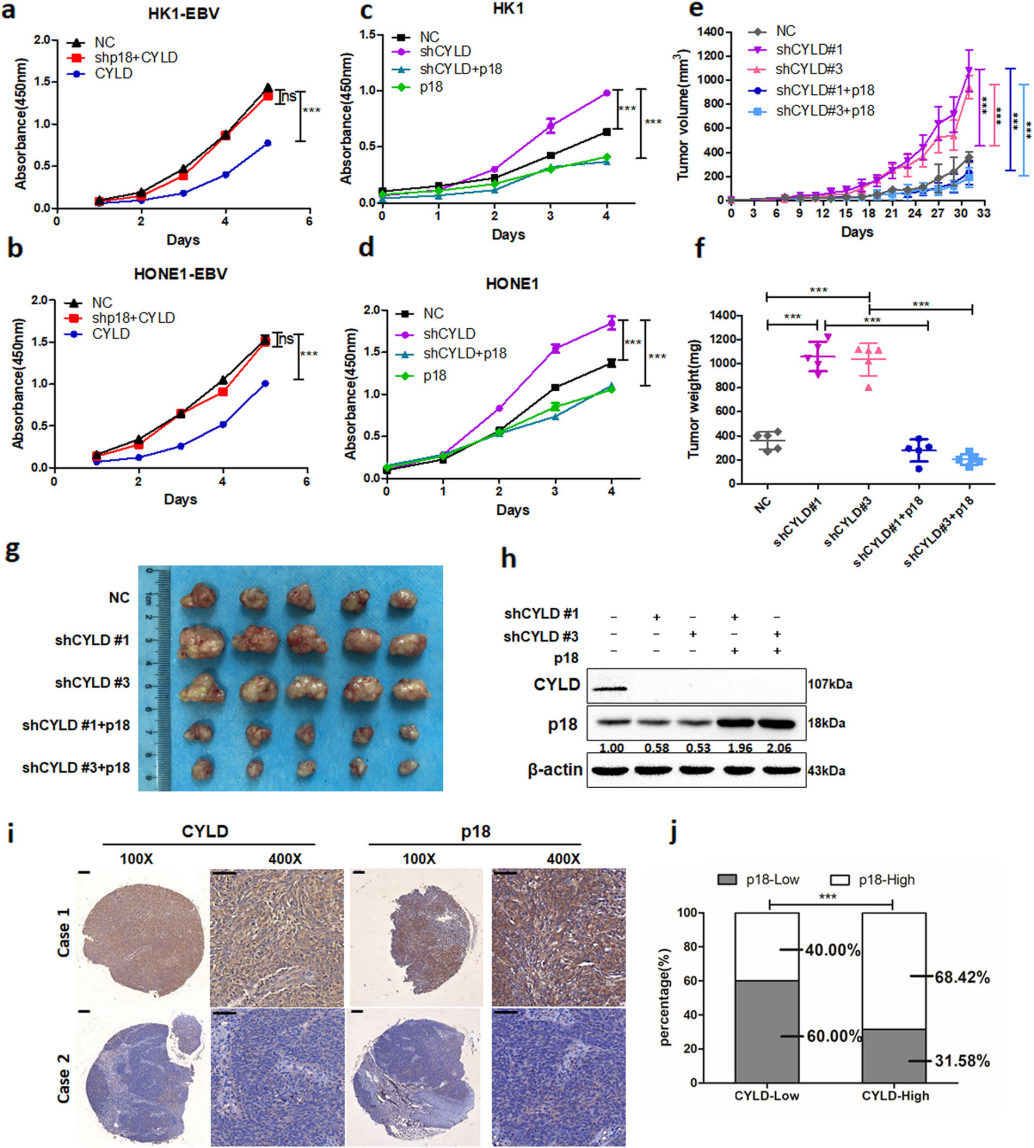
CYLD functions as a tumor suppressor by regulating p18. **a**–**d** Cells were infected with the lentiviral shRNAs and then infected with the indicated plasmids. Cell proliferation was monitored using CCK8 assays with cells harvested at the indicated time points. Statistical significance was determined by a two-tailed, unpaired Student’s t test. The indicated groups of cells (5 × 10^6^) were subcutaneously injected into mice. **e** Tumor growth, **f** tumor weight, and **g** tumor images are shown. **h** HONE1 cells implanted into nude mice were disrupted and analyzed by Western blotting. **i** Representative IHC staining of CYLD and p18 expression from a tissue microarray of nasopharyngeal squamous cell carcinoma patients (100×: scale bar, 100 μm; 400×: scale bar, 50 μm). **j** The p18 protein expression level was calculated according to CYLD expression in a tissue microarray of nasopharyngeal squamous cell carcinoma patients. High and low groups were according to median expression. (Data are represented as mean ± SEM (*n* = 3). Differences were considered significant at ***p < 0.001).

To investigate whether CYLD affects cell proliferation through p18, we conducted a cell proliferation assay using CCK8. In p18 knockdown HK1-EBV and HONE1-EBV cells, overexpression of CYLD didn’t inhibit cell proliferation (Fig. 7a, b). In addition, CYLD knockdown promoted the proliferation of HK1 and HONE1 cells, and restoration of p18 completely reversed the effect of CYLD depletion (Fig. 7c, d). To further confirm the role of CYLD in NPC cells in vivo, stable CYLD knockdown HONE1 cells were implanted into nude mice. Compared with mice implanted with control shRNA infected cells, the tumor volume and tumor weight were significantly increased in mice injected with CYLD knockdown HONE1 cells. Importantly, restoring p18 expression fully reversed the tumor-promoting effect of *CYLD* shRNA (Fig. 7e–g). Tumor protein analysis by Western blot also showed that the effect of CYLD depletion on p18 was retained in these tumors (Fig. 7h). Overall, our data demonstrate that CYLD exerts a p18-dependent tumor-suppressing function. Next, to evaluate the relevance between CYLD and p18 in NPC, IHC staining of p18 and CYLD was conducted in a NPC tumor tissue microarray. Among the 44 NPC specimens, a significantly positive correlation calculated by the Pearson χ2 test was observed between the two proteins (*r* = 0.51, *p* < 0.001, Supplementary Table 6), the percentage of high p18 IHC scores in specimens having high expression was 68.42% (Fig. 7i, j). Thus, these results suggest that p18 is a tumor suppressor target of CYLD in NPC.

## Discussion

**Fig. 8:**
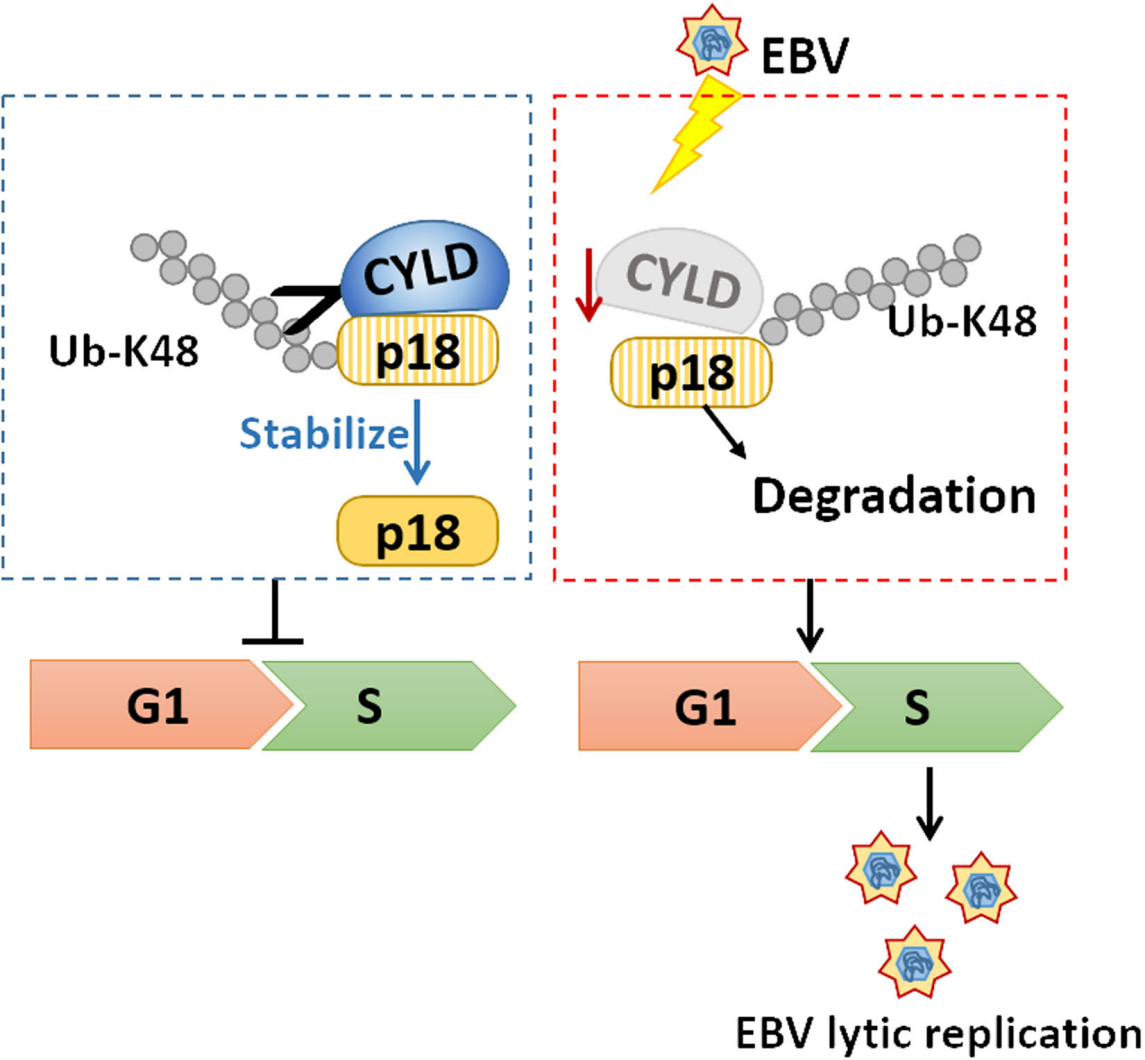
Pattern diagram showing that stabilization of p18 by CYLD is pivotal for cell cycle progression and viral replication. Deubiquitination and stabilization of the p18 protein by CYLD delays the G1/S transition. Reduced expression of CYLD in NPC cells by EBV wrecks CYLD-p18 axis, which providing a replication passive environment for EBV lytic replication.

In this paper, we identified CYLD as a deubiquitinase of CKI p18, offering mechanistic insights into the contribution of CYLD-p18 axis to the cell cycle progression. Reduced expression of CYLD in NPC cells by EBV provides a replication passive environment and results in increased EBV lytic replication (Fig. 8). These findings provide an avenue for further understanding the CYLD function in oncogenesis.

CKIs are key negative regulators of cell cycle progression. Post-translational modification mechanisms involving the ubiquitin-proteasome pathway are the dominant mechanisms in regulating protein abundance of CKIs, and the same is true of p18^34^. However, so far no post- translational regulator, especially DUBs, has been identified to regulate the protein stability of p18. As for CYLD, studies have shown that CYLD binds to and deubiquitylates BCL-3 inhibiting its nuclear translocation, leading to decreased transcription of CCND, and delayed cells from entering S-phase^35^. However, no direct target of CYLD has been identified in cell cycle. In the current work, we found that CYLD mainly prevents the G1/S cell cycle progression, but no significant change occurred in cyclin D or CDK4 and 6. Because of the important role of CKIs in cell cycle progression by inhibiting CDK-cyclin complex, we examined the CKIs role in G1/S cell progression, and found that CYLD specifically stablized the protein level of p18 by interacting and deubiquitating p18. Further, PLA assay results showed the interaction between CYLD and p18 is by direct binding. Recovery of p18 in CYLD knockdown cells prevents the G1/S transition induced by CYLD knockdown, indicating that CYLD exerts its function through p18.

CYLD has been extensively confirmed as a DUB specific removing K63/M1 ubiquitylation chains^36^. Recent studies have found that that CYLD can also cleave K48 ubiquitin chains in directly or indirectly ways^37–39^. In this work, we found CYLD regulates the p18 expression in protein level, and ubiquitylation assay showed CYLD specifically removing K48 ubiquinatin chains on p18, without level changing of K63 and other types of ubiquinatin chains. Which indicating that CYLD directly interacts and deconjugates K48 ubiquitin chains from p18 to maintain its expression. Our study provides some evidence as to CYLD can remove K48 ubiquitin chains from substrates.

CYLD works as a tumor suppressor in multiple cancers^40,41^. Downregulation of CYLD is correlated with tumor initiation, development, metastasis, and resistance to treatment^16,42–44^.However, the expression and function of CYLD in NPC is still unclear. Combined with data base and NPC tissues microarray, we found a low level of CYLD in NPC and associated with poor prognosis. Knockdown of CYLD resulted in increased proliferation and overexpression of p18 reversed this tumor-promoting effect in vitro and in vivo, indicating that CYLD exerts its tumor suppressor function dependent on p18.

In our findings, we identified that EBV infection as an important cause of CYLD downregulation through using clinical samples and cell line analysis. And recovery of CYLD decreased EBV- associated tumor growth in vitro and vivo. While the mechanism of CYLD down-regulation by EBV is still unclear. As a next step, we will further investigate the mechanisms of EBV in regulating CYLD. EBV infection confers in vivo proliferative advantage to infected-NPC cells^26^. Overexpression of CYLD in EBV ( + ) NPC cells markedly inhibited tumor growth in athymic nude mice. EBV altered the growth properties of infected cells by breaching the CYLD-p18 axis providing insights into the role of EBV in the pathogenesis of human epithelial cancers. Tuning of the cellular environment, together with the expression of EBV tumorigenic EBV genes, jointly promote tumorigenesis. Restoring CYLD represents potential therapeutic strategies for treatment of EBV-positive malignancies.

Accumulating evidence indicates that inducing an S-phase-like environment is necessary for efficient replication of the viral genome^45^. EBV disturbs host signaling pathways, which result in both beneficial and detrimental effects on viral replication or oncogenesis^46–48^. Notably, a recent study showed that gamma herpes virus encoded viral cyclin to functionally bypass the cyclin-dependent kinase inhibitor p18, leading to viral lytic reactivation^49^. EBV belongs to gamma herpes virus IV, and doesn’t encode viral cyclin, which means that EBV has to utilize host mechanisms to up-regulate S-phase cyclins. In our cell models, expression of CYLD extensively inhibited EBV lytic replication. As we found that CYLD is a deubiquitinase of p18 in regulating G1/S transition, which means that the CYLD-p18 axis may prevent EBV lytic replication by regulating cell cycle procession. Expression of CYLD in p18 knockdown HONE1-EBV cells had no effect on EBV lytic replication. In the present study, we showed that EBV bypasses p18 by depressing its deubiquintase activity. Therefore, we concluded that EBV deregulates the host CYLD-p18 axis to push host cells to provide suitable replication-passive cellular environments for EBV DNA replication.

## Methods

### Cell culture

The human NPC cell lines, HK1, HK1-EBV, HONE1, and HONE1-EBV, were generously provided by Professor Sai Wah Tsao from the University of Hong Kong. Cells were cultured in RPMI-1640 medium (Cat: 11875500, Gibco, Grand Island, USA) supplemented with 10% fetal bovine serum (FBS; Cat: 04-001-1, BI, Kibbutz Beit-Haemek, Israel). The immortalized human nasopharyngeal epithelial cell lines, NP460hTERT and NP460hTERT-EBV, were generously provided by Professor Sai Wah Tsao from the University of Hong Kong.Cells were cultured in a 1:1 mixture of Defined Keratinocyte-SFM and EpiLife medium (Cat: 10744019 and MEPI500CA, Gibco). Cells were cultured in 37 °C incubators with 5% CO_2_.

### Reagents and antibodies

MG132 (Cat: HY-13259) and the protease inhibitor cocktail (Cat. HY-K0010) were purchased from MCE (New Jersey, USA). LipoMax plasmid transfection reagent (Cat. 18101223) was purchased from SUDGEN (Bellevue, WA, USA). Dynabeads (Cat. 10002D) were purchased from Thermo Fisher (Waltham, MA, USA) and IP buffer (Cat. P0013) was purchased from Beyotime (Shanghai, China). Cell Counting Kit-8 solution (CCK-8) was purchased from DOJINDO (Cat. CK04, Kyushu, Japan). Anti-mouse IgG-HRP (1:3000, Cat. SC-2005) and anti-rabbit secondary antibody (1:3000, Cat. SC-2004) were purchased from Santa Cruz BioTechnology (California, USA). Anti-CYLD antibody (1:1000, Cat: 8462 S) and mouse species anti-p18 (1:1000, Cat: 2896 S) were purchased from Cell Signaling Technology (Boston, USA). Primary antibodies include anti-p18 INK4C (1:1000, Cat: ab192239), and anti-p19 (1:1000, Cat: ab80), p15 (1:1000, Cat: ab53034), p16 (1:1000, Cat: EPR20418), p21 (1:1000, Cat: ab109520), p27 (1:1000, Cat: ab32034), and anti-Ub (1:1000, ab134953) were purchased from Abcam. Anti-Flag antibody was purchased from Sigma Aldrich (1:1000, Cat: F3165). Anti-Myc antibody was purchased from Cell Signaling Technology (1:1000, Cat: 3946 and 2276). Anti-RIP1 antibody was purchased from BD (1:1000, Cat: 610458, New Jersey, USA). Anti-GST antibody was purchased from Cell Signaling Technology (1:1000, Cat: 2624). EBV relative primary antibodies included anti-Zta antibody (1:200, sc-53904, Santa Cruz BioTechnology), anti-EAD antibody (1:1000, MAB8186, Merck Millpore, MA USA). All antibodies were used according to the dilution ratio in the instructions.

### Plasmids and lentivirus transduction

The full length and deletion constructs of CYLD and p18 were synthesized by GeneChem (Shanghai, China). The PLVX-CYLD plasmid was purchased from Tsingke (Beijing, China). The PHBLV-p18-plasmid was purchased from Hanbio (Shanghai, China) and shRNA targeting CYLD lentiviruses was purchased from GenePharma (Shanghai, China). The shRNA target sequences used included: CYLD#1, GCGTGTGTTGAAAGTACAATT; and CYLD#3, GCTGTAACTCTTTAGCATTTG. Target cells were infected with lentivirus for 24–48 h according to the manufacturer’s instructions. The shRNA targeting p18 lentiviral plasmids was purchased from ZORIN (Shanghai, China) The GST-CYLD plasmid was purchased from GeneChem. The wild type HA-ubiquitin plasmid and mutant HA-ubiquitin plasmids (K6, K11, K27, K29, K33, K48, and K63) were gifts from Prof. Hui Zheng (Soochow University, Suzhou, Jiangsu).

### RNA isolation and real-time PCR

Total mRNA was isolated by using the NucleoZOL reagent (Cat: 740404, MACHEREY- NAGEL GmbH & Co. KG, Düren, Germany). The RevertAid First Strand cDNA Synthesis Kit (Cat: K1622, Invitrogen, Carlsbad, CA, USA) was used for reverse transcription. Real-time PCR analysis was performed in triplicate using the SYBR™ Green Master Mix (Cat: A25742, Invitrogen) and the ABI7500 Real-Time System (Applied Biosystems). All primers used in this study were list in Supplementary Table 7.

### Immunohistochemistry

Nasopharyngitis and NPC tissues were collected from the Department of Pathology at Xiangya Hospital, Central South University, and Changsha, China. All patients signed informed consent forms for sample collection. The study was approved by the Medical Ethics Committee of Xiangya Hospital, Central South University (No. 201803134). The NPC tissue array (*n* = 70) was purchased from Pantomics (Richmond, CA, USA). The NPC tissue array (*n* = 129) was purchased from Outdo Biotech (HNasN129Su01, Shang Hai, China). CYLD, p18 or EAD antibody was applied overnight in 4 °C at a dilution of 1:100. Then slides were then incubated with secondary antibody. After staining with DAB solution (Abcam), re-staining with hematoxylin. Slides were scanned by Pannoramic MIDI digital slide scanner (3DHISTECH). The staining score of each protein was analyzed by two pathologists independently. The immunohistochemistry score depended on the degree of staining and the rate of positive cells. The patients were divided into low and high expression groups according to the median scores.

### Protein half-life assays

Cells were treated with cycloheximide (20 μg/mL) for various periods to block protein synthesis. Protein levels were measured by Western blot analysis.

### Western blotting

Cells were harvested and disrupted in IP lysis buffer with protease inhibitor (HY-K0010, MCE), for 30 min on ice, and centrifuged for 15 min at 12,000*g* at 4 °C. 50 μg of protein were separated by 12% SDS-PAGE and transferred onto PVDF membranes (Millipore). Membranes were blocked with 5% nonfat milk (or 5% BSA) for 1 h at room temperature, and then incubated with the specific primary antibody at 4 °C overnight. And then with HRP-conjugated anti-mouse or anti-rabbit secondary antibody. Visualization was performed using the ChemiDoc XRS system and Image Lab software (Bio-Rad, CA, USA). Western blots were derived from the same experiment and processed in parallel. Uncropped scans of the Western blots are provided in Supplementary materials.

### DUB activity measurement

CYLD activity was determined by Deubiquitinase Activity Assay Kit (Catalog # K485-100, Biovision, CA, USA). 3 × 10^7^ cells were washed with ice cold PBS and add 300 μl ice-cold DUB assay buffer with 1 mM DTT, which was provided by kit. Then centrifuged for 15 min at 12,000 *g* at 4 °C. The separation method of CYLD refers to the previous reports^50^. The specific steps are as follows. The sample was pre-cleared using 30 μl of Dynabeads protein A (Cat: 10004 A, Invitrogen, MA, USA) for 1 h at 4 °C. Centrifuge lysate (tissue or cell) at 10,000 *g* for 5 min. at 4 °C. Collect the supernatant. Then cleared cell lysates were incubated with Protein A beads pre-loaded with 2 mg of anti-CYLD antibody for 1 h. The preparation of Ab-conjugated Dynabeads were according to the manufacturer’s instructions of Invitrogen. After incubation, beads were washed twice with IP lysis buffer containing cocktail. Resuspending beads in 50 μl DUB assay buffer (1 mM DTT). 5 μl of beads from immunoprecipitation was analyzed by Western blotting with anti-CYLD antibody to determine relative levels of CYLD. Add 10 μl into a well of the provided half-area 96-well plate. Detection of fluorescence value by multi-functional enzyme labeling instrument (PerkinElmer VICTOR™ X3, USA). Measure fluorescence (Ex/Em = 350/440 nm).

### Cell proliferation assay

For the viability assay, cells were cultured in 96-well plates and viability was examined using the CCK-8 solution according to the manufacturer’s instructions. The ratio of culture medium to CCK-8 solution was 9:1, 100 μl mixed solution was added to each well, and 450 nm absorbance was detected after 30–60 min in 37 °C, 5% CO_2_. The plate was shaken evenly and the color intensity was read at 450 nm wavelength on a microplate reader (Biotek EL × 800, USA).

### Tumorigenicity assay

Tumorigenicity study was approved by the Medical Ethics Committee (for experimental animals) of Xiangya Hospital, Central South University (No. 201803135). Then 4 × 10^6^ cells per animal were injected subcutaneously into the flank regions of nude mice (BCLB/c-nu, female, 5 weeks old). The tumors were measured every other day and tumor volume was calculated using the following formula: V = (π × length × width^2^)/6. At the end of experiments, mice were euthanized by CO_2_ inhalation, and the weight of extracted xenograft tumors was obtained at the same time.

### Cell cycle analysis

Cells stably expressing the indicated plasmids were incubated in serum-free culture medium for 24 h, and then harvested with serum-containing medium after another 4 h. The cells were gently fixed with 70% ethanol at 4 °C overnight. Thereafter, the cells were incubated with RNase for 30 min at room temperature in the dark, then stained with propidium iodide, and cellular fluorescence was measured using BD accuriC6 (BD Biosciences, San Jose, USA). Data were analyzed by flow cytometry™ softer ware version 10.

### Edu assay

Cells stably expressing the indicated shRNAs were cultured in 96-well plates and examined using the EdU Cell Proliferation Kit with Alexa Fluor 647 from BeyoClick (Cat: C0081L, China) according to the manufacturer’s instructions. The specific steps are as follows. The Edu working fluid was prepared with fresh complete RPMI-1640 medium at a concentration of 20 μM. The 2X Edu working fluid was preheated at 37 °C and added to a 96-well plate in equal volume and incubated at 37 °C, 5% CO_2_ for 2 h. Next, cells were fixed with 4% paraformaldehyde for 15 min and permeated with 0.3% TritonX-100 for another 15 min at room temperature. Each well was incubated with 60 μL click additive solution for 30 min at room temperature in the dark. Cell nuclei were stained with Hoechst 33342 for 10 min to detect. The fluorescence was measured by Operetta CLS CellInsight™ CX5 imaging system (Perkin Elmer, USA), and the data were analyzed using Harmony 4.5 software. Average Edu intensity greater than 500 is considered Edu positive. The ratio of Edu-positive cells to total cells is the Edu-positive percentage^51^.

### Co-immunoprecipitation

Cells were disrupted in IP lysis buffer containing protease inhibitor cocktail and 1 mg protein aliquots were pre-cleared by incubating with 10 μL Dynabeads protein A (Invitrogen) for 1 h at 4 °C. The pre-cleared samples were incubated with antibody (2 μg/sample) overnight at 4 °C and then 20 μL Dynabeads protein A were added to samples and incubated for 2 h at 4 °C. The beads were washed 3 times with cold wash buffer, then boiled with 1X loading buffer and analyzed by Western blotting.

### Immunofluorescence analysis

Cells were washed with complete medium, and fixed in 3.7% formaldehyde at 37 °C for 20 min, permeabilized for 15 min with 0.1% Triton X-100. Then the cells were blocked in 5% donkey serum for 1 h at room temperature and incubated with a primary antibody in PBS with 1% BSA at 4 °C overnight. Cells were washed with 1 × PBS and incubated with a secondary antibody for 45 min. Then cells were washed and stained with DAPI for 10 min and viewed by a confocal microscope (Leica TCS SP8, Germany).

### Proximity ligation assay

Interacting proteins were detected by the DuoLink® In Situ Red Starter Kit Mouse/Rabbit (DUO92101, Sigma-Aldrich, Darmstadt, Germany). Cells were seeded in 8-well chamber slides (Millicell EZ SLIDE, Millipore, Darmstadt, Germany) and cultured overnight. Slides were washed with cold 1 × PBS and fixed in 4% paraformaldehyde for 30 min, then blocked with Duolink Blocking Solution in a pre-heated humidified chamber for 30 min at 37 °C, and then permeabilized in 0.1% Triton X-100 for 20 min^47^. The primary antibodies to detect CYLD and p18 were added to the slides and incubated overnight at 4 °C. On the following day, slides were incubated with the PLA probes diluted 1:5 in antibody diluents in a pre-heated humidity chamber for 1 h at 37 °C. Subsequent steps were performed according to the manufacturer’s protocol. Fluorescence images were acquired using a Leica TCS SP8 confocal microscope.

### In vivo ubiquitination assay

Ubiquitination assays were performed as described^52^. The specific steps are as follows. For in vivo ubiquitination assay of p18, 293 T cells were transfected with Myc-p18, HA-Ub (WT), or HA-Ub mutants and Flag-CYLD for 48 h followed by treatment with 20 μM MG132 for 6 h. Cell were disrupted in 1% SDS lysis buffer containing protease inhibitors for 50 min, then cell lysates were heated at 95 °C for 10 min and diluted 10 times in non-SDS bufferand samples were incubated at 4 °C for 30 min with rotation. Then, the lysates were centrifuged to obtain the cytosolic protein fraction. Myc-p18 was immunoprecipitated using anti-Myc at 4 °C overnight before incubation with protein A beads. Samples were washed and prepared for immunoblot analysis as described above.

### In vitro ubiquitination assay

293 T cells weretransfected with Myc-p18 with or without cotransfected HA- ubiquitin and treated with 20 μM MG132 for 6 h. Nonubiquitylated or ubiquitylated Myc-p18 was purified from the cell extracts using an anti-Myc antibody. Either GST-tagged CYLD was expressed in the BL21 Escherichia coli strain. Recombinant CYLD protein purified according to instructions of GST Labeled Protein Purification Kit (Cat.P2262, Beyotime, Shanghai, China). Nonubiquitylated or ubiquitylated Myc-p18 was incubated with purified CYLD in deubiquitylation buffer (50 mM Tris·HCl, pH 8.0, 50 mM NaCl, 1 mM EDTA, 10 mM DTT, and 5% glycerol) for 2 h at 37 °C.

### DNA extraction and EBV DNA copy detection

Total DNA from cells and culture supernatant fractions was extracted using the QIAamp DNA Mini Kit (Cat: 51304, QIAamp), following the protocol recommended by the manufacturer. A final elution volume of 200 μl was obtained after the extraction procedure was complete. DNA concentration was quantified using a NanoDrop 1000 Spectrophotometer (Nano-Drop Technologies, Waltham, MA, USA). EBV DNA copies were detected using the EBV nucleic acid quantitative detection kit, which targets the BamHI-W region of the EBV DNA genome.

### Database analysis

The GEO database can be accessed online (https://www.ncbi.nlm.nih.gov/geo/). GSEA (Gene Set Enrichment Analysis) was performed by using 31 NPC data sets (GSE12452). TCGA database (*N* = 283) was used to examine the overall survival of patients according to CYLD levels in head and neck squamous cell carcinoma.

### Statistical analysis

The experimental results were statistically evaluated using the Student’s *t* test, the Pearson chi-square test, ANOVA, COX regression analysis, and Kaplan–Meier analysis. A value of *p* < 0.05 was considered statistically significant. All statistical analyses were performed using SPSS 17.0 or Graphpad prism 5.0 software.

## Supplementary information

Supplementary materials

nr-reporting-summar

## Data Availability

The data generated and analysed during this study are described in the following data record: 10.6084/m9.figshare.13536902^53^. The histology images and patient survival data are not publicly available for the following reason: data contain information that could compromise research participant privacy. However, the data can be made available upon reasonable request to the corresponding author. The NPC patients CYLD expression data used in the study can be accessed via Oncomine (https://www.oncomine.org/) via the following process. First, sign in with a registered mailbox. Then search with Gene: CYLD; Analysis Type: Cancer vs. Normal Analysis; Cancer Type: Head and Neck Cancer. Log-in is required to access Oncomine data. The data are freely available for academic and non-profit communities. The HNSCC patient survival data used in the study are stored in the Excel file ‘HNSCC patient survival data.xlsx’ and are publicly available via The Cancer Genome Atlas (TCGA). SurvExpress (http://bioinformatica.mty.itesm.mx:8080/Biomatec/SurvivaX.jsp) was used to analyse the data on TCGA, and Internal ID: 100. The GSEA data used in the study are openly available via the Gene Expression Omnibus repository via accession https://identifiers.org/geo:GSE12452^54^. The reagents and other materials (except for the received cells and plasmids) are available from the corresponding author upon request.
