# Supplementary material for: Stabilization of p18 by deubiquitylase CYLD is pivotal for cell cycle progression and viral replication
Source: NPJ Precis Oncol. 2021 Mar 2;5:14. doi: 10.1038/s41698-021-00153-8 (PMC7925679; doi:10.1038/s41698-021-00153-8)

Supplementary materials

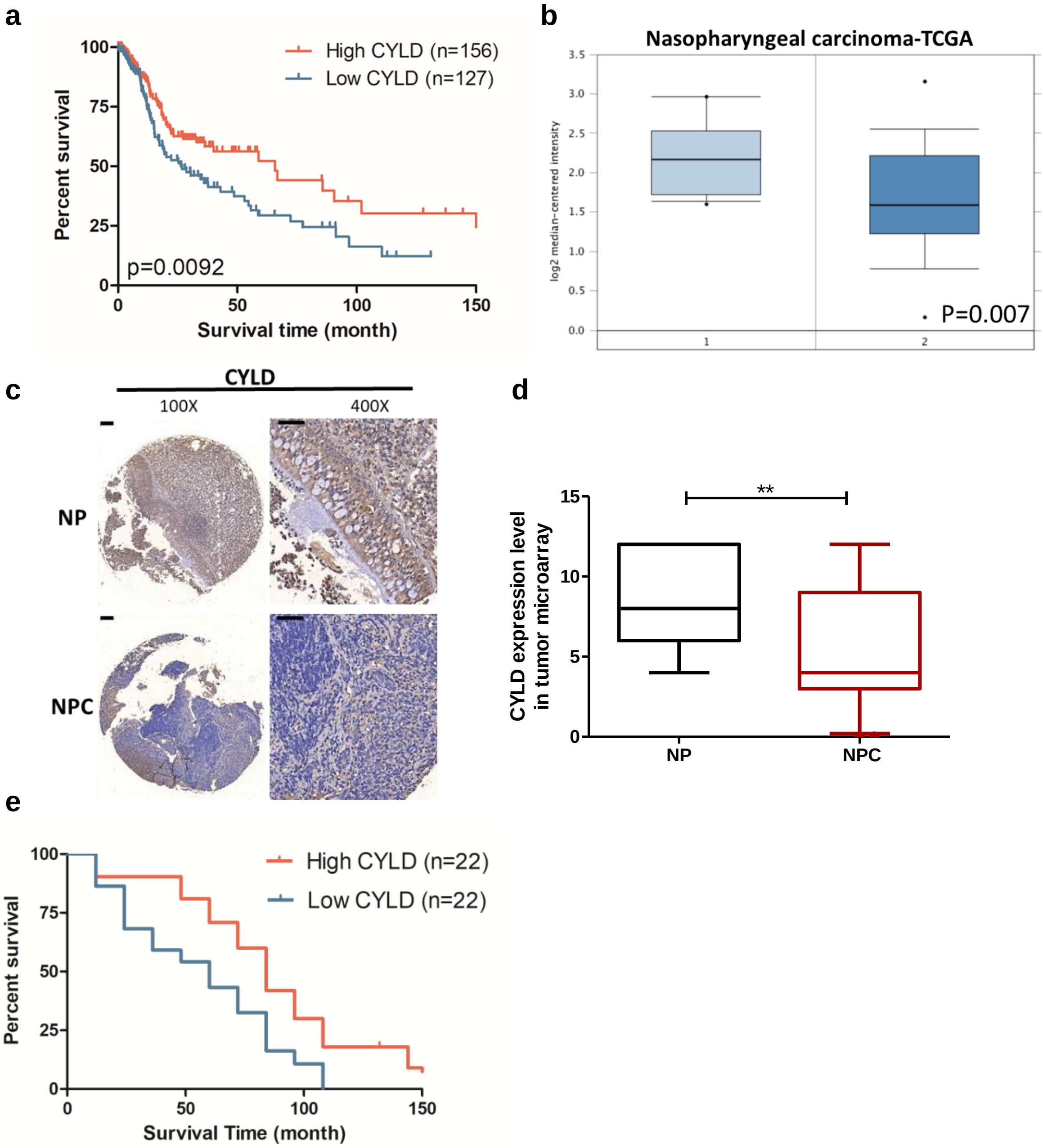

Supplementary Figure 1. CYLD is downregulated in NPC

a Overall survival rates of HNSC patients (TCGA database) with low (n = 127) or high (n = 156) mRNA levels of *CYLD* were estimated by the Kaplan–Meier method using a log-rank test (p = 0.0092). Group according to CYLD median expression. **b** Downregulation of CYLD expression in NPC compared to nasopharynx (Oncomine). **c** Representative IHC staining of CYLD expression from a tissue microarray of NPC patients (100×: scale bar, 100 μm; 400×: scale bar, 50 μm). **d** Histoscores of CYLD expression from the tissue microarray. NPC patients (n = 44) and nasopharyngitis (NP) tissue (n = 15). **e** Overall survival rates of NPC patients with low (n = 22; median survival time: 48 months) or high (n = 22; median survival time: 72 months) expression levels of CYLD were estimated by the Kaplan–Meier method using log-rank test. Group according to CYLD median expression.

a

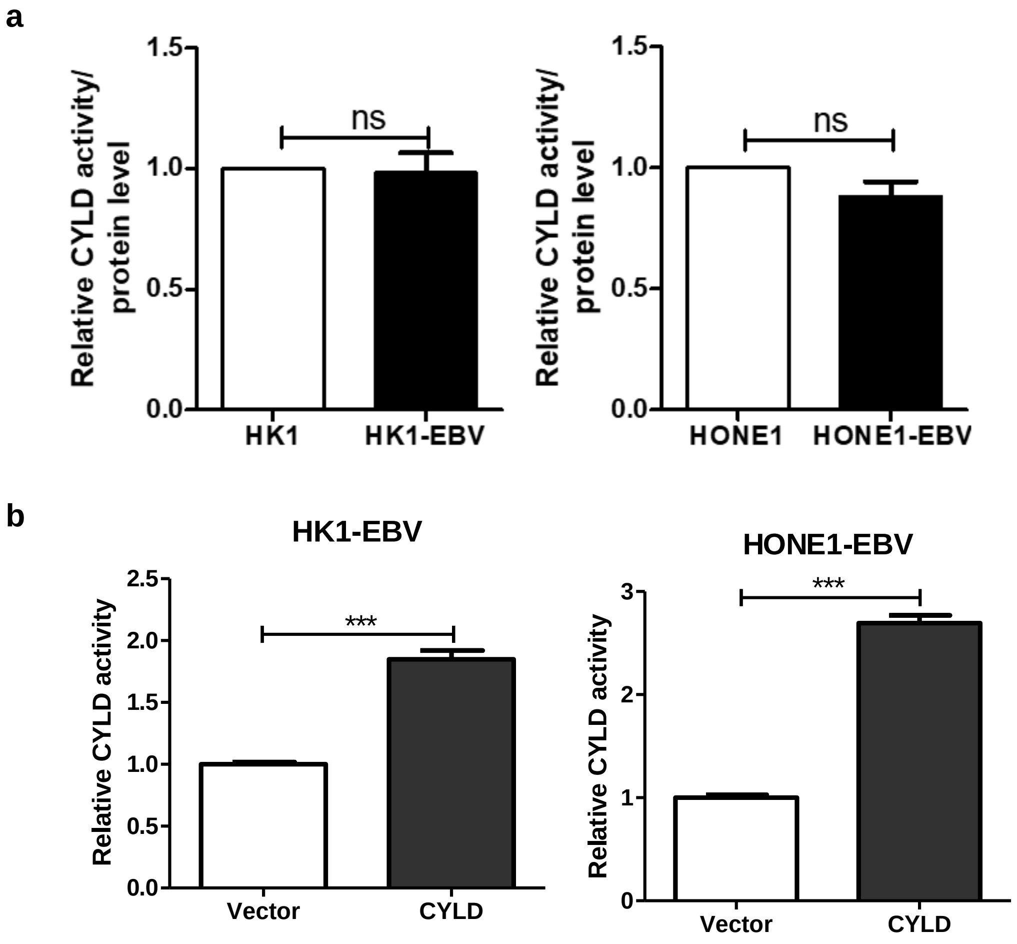

Supplementary Figure 2. Decrease of CYLD activity is caused by expression inhibition

a DUB activity measurement of CYLD is normalized with CYLD protein level. **b** DUB activity measurement of CYLD in CYLD overexpressed HK1-EBV and HONE1-EBV cells. (Values represent means ± S.E.M. of 3 independent experiments performed; *p＜ 0.05, **P＜0.01, ***P＜0.001).

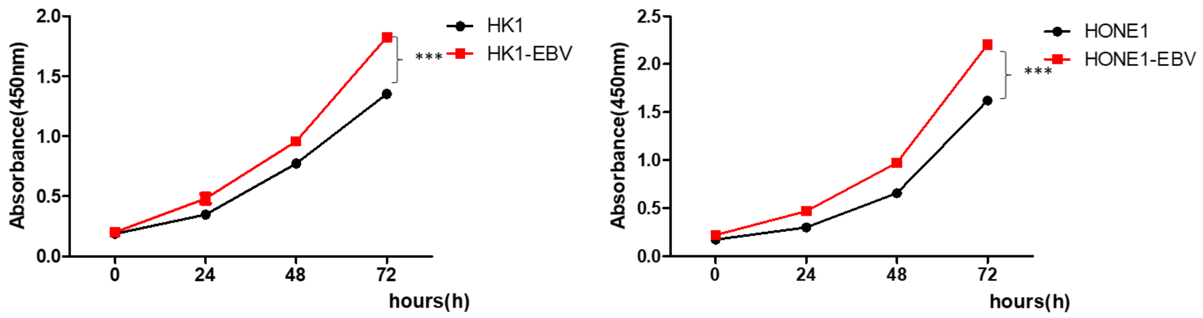

Supplementary Figure 3. EBV promotes cell proliferation

Cell proliferation was monitored using CCK8 assays with cells harvested at the indicated time points. Statistical significance was determined by a two-tailed, unpaired Student's t test. (***P＜0.001).

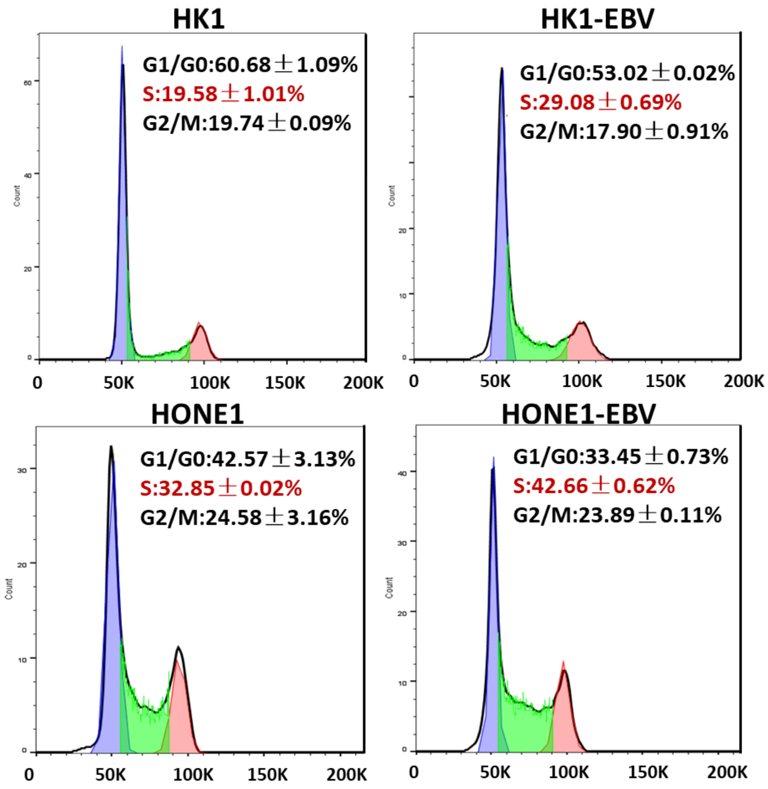

Supplementary Figure 4. EBV contributes to cell G1/S transition.

HK1-EBV and HONE1-EBV cells was examined by staining with propidium iodide and analyzed by flow cytometry.

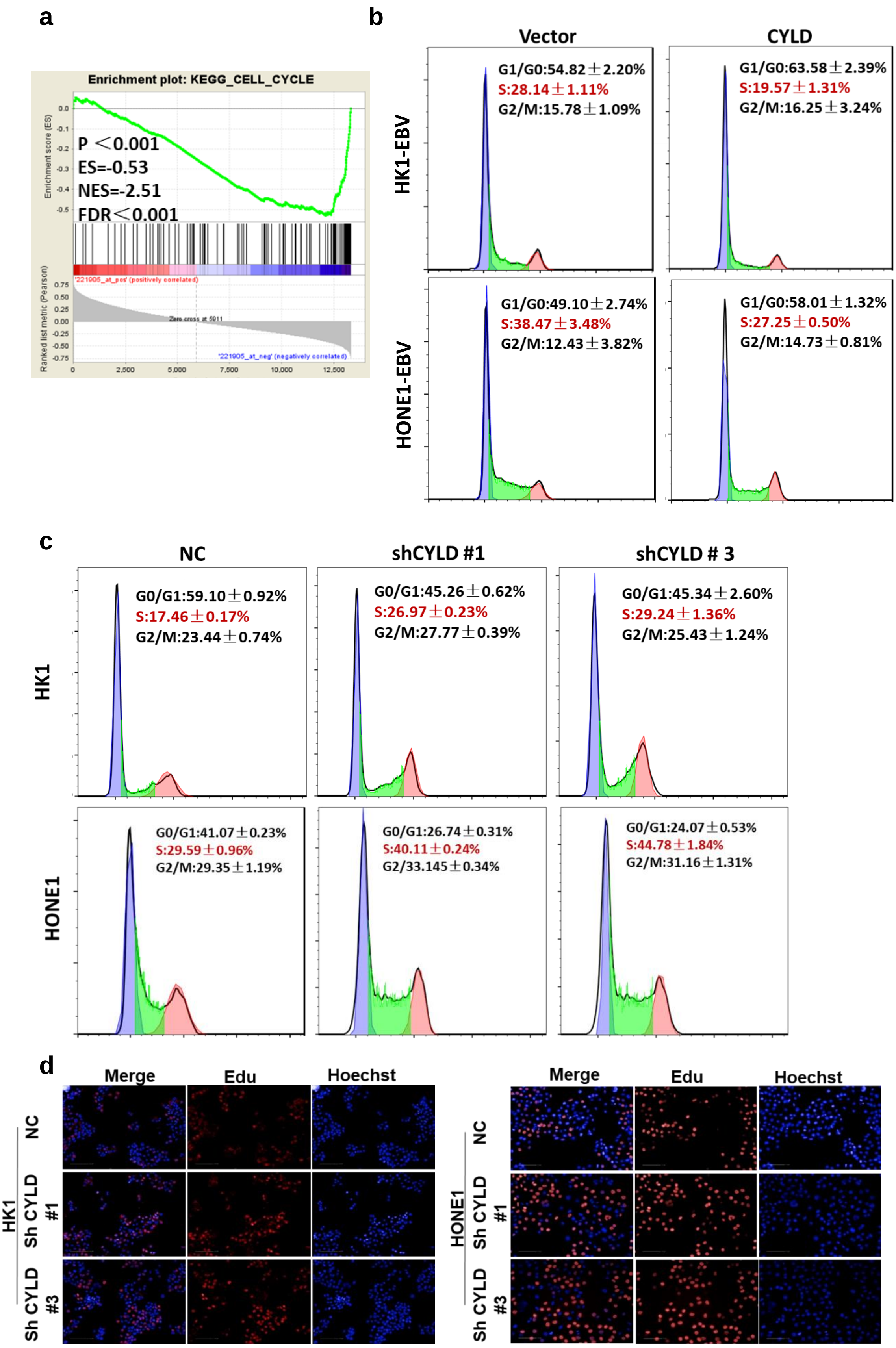

Supplementary Figure 5. CYLD inhibits the G1/S cell cycle transition

a Analysis of CYLD-related signaling pathways in 31 EBV (+) NPC samples by GESA (GES12452). **b** HK1-EBV and HONE1-EBV cells infected with the CYLD plasmids were stained with propidium iodide and analyzed using flow cytometry. **c** Knockdown of CYLD in HK1 and HONE1 cells was examined by staining with propidium iodide and analyzed by flow cytometry. **d** S-phase cell was analyzed by Edu staining. Average Edu intensity above 500 is considered positive. Nuclei were stained with Hoechst. (Scale bar=100 μm).

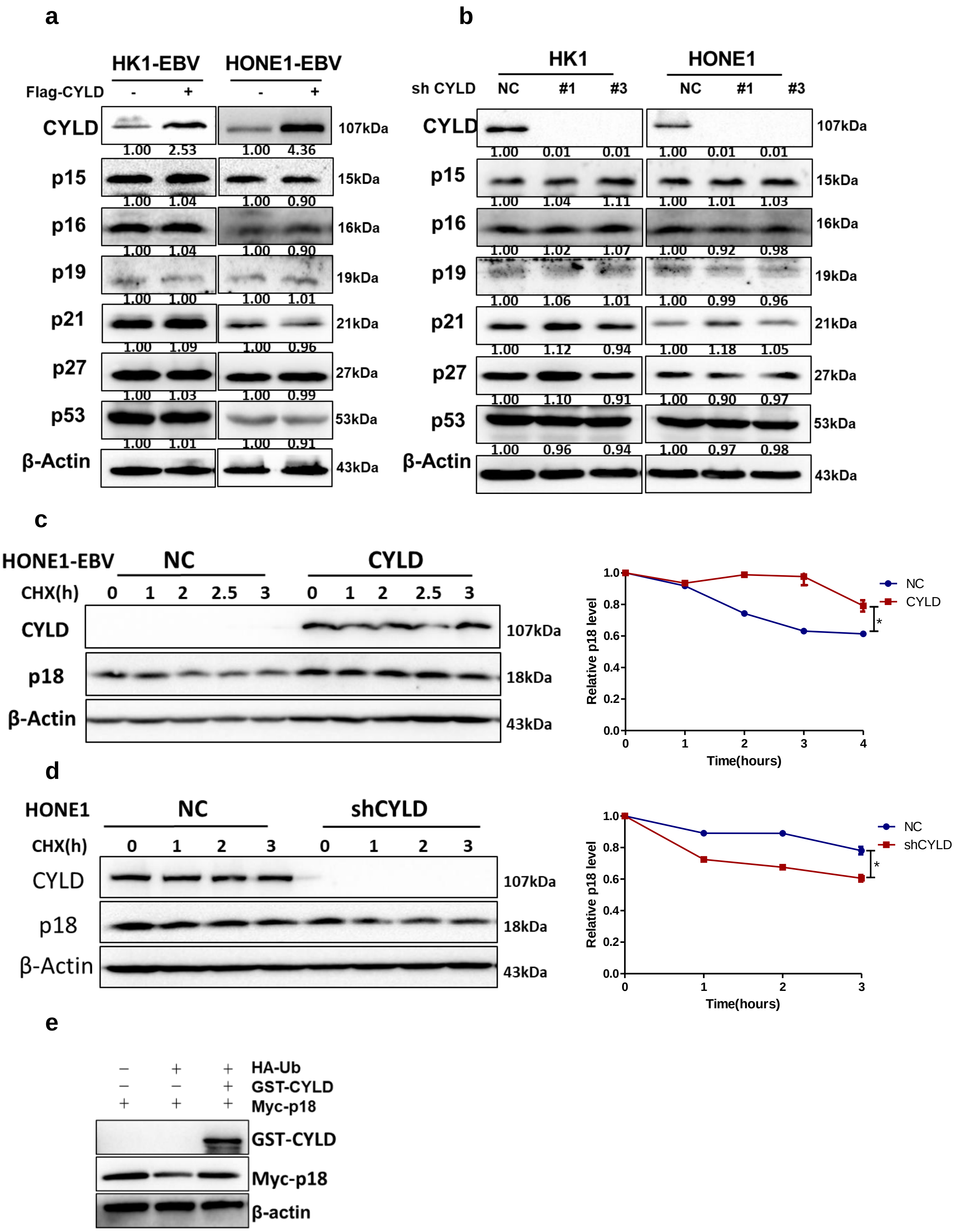

Supplementary Figure 6. CYLD regulates the protein level of p18.

a HK1-EBV and HONE1-EBV cells transfected with CYLD were immunoblotted with antibodies against the indicated proteins. **b** HK1 and HONE1 cells were infected with

CYLD lentiviral shRNAs and cell lysates were immunoblotted with antibodies against the indicated proteins. **c** HONE1-EBV cells transfected with the indicated constructs were treated with 20 μg·mL^{-1} CHX, collected at the indicated time points, and immunoblotted with anti-CYLD, anti-p18, or anti-β -actin. Quantification of the p18 levels relative to β-actin expression is shown. **d** HONE1 cells transfected with the indicated lentiviral shRNAs were treated with 20 μg·mL^{-1} CHX, collected at the indicated time points, and immunoblotted with anti-CYLD, anti-p18, or anti-β-actin. Quantification of the p18 levels relative to β-actin expression is shown. **e** Ubiquitylated Myc-p18 was incubated with GST-tagged CYLD or not. After coincubation, Myc-p18 was immunoprecipitated using an anti-Myc antibody, and analyzed using SDS/PAGE. (*$p < 0.05$).

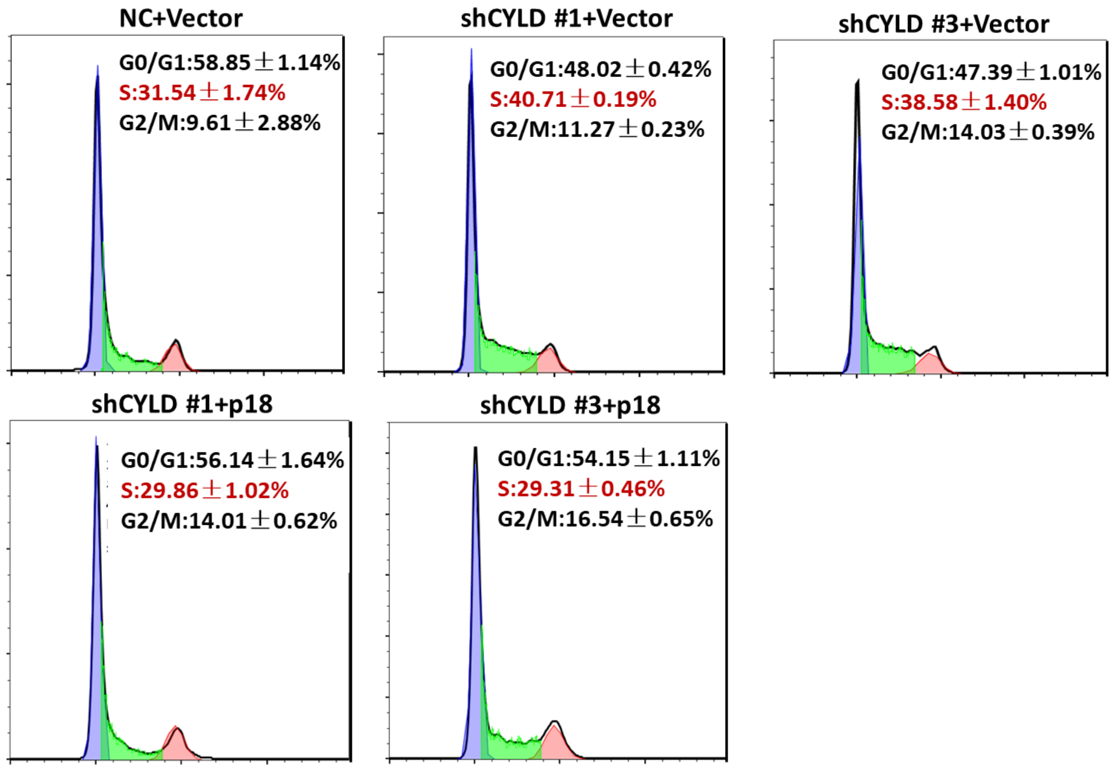

Supplementary Figure 7. CYLD affects cell cycle progression through p18.

HONE1 cells were infected with the indicated lentiviral shRNAs followed by transfection with the indicated constructs, then stained with propidium iodide and analyzed by flow cytometry.

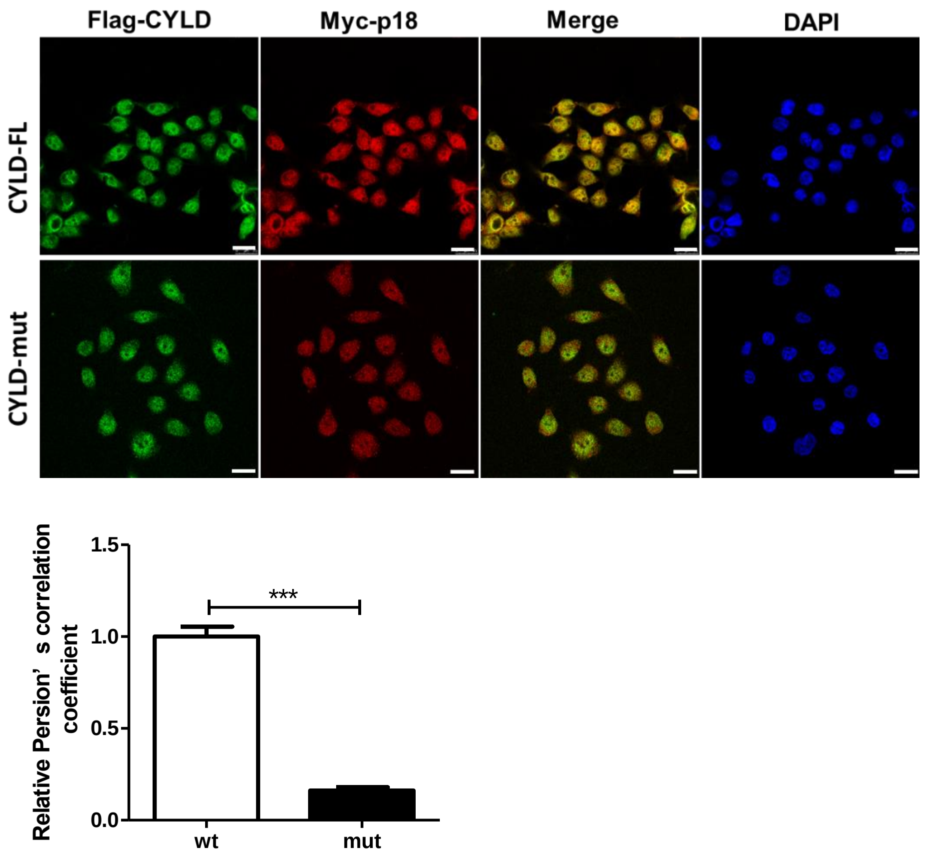

Supplementary Figure 8. CYLD interacts to p18 through its N-domain.
Flag-tagged full length (CYLD-FL) or N-domain deleted mutant (CYLD-mut) CYLD plasminds together with Myc-p18 were transfected into HONE1 cell. Flag-CYLD (green) and Myc-p18 (red) in HONE1 cells was visualized by immunofluorescence with anti-CYLD and anti-p18. DNA was stained with DAPI, and a merged view of the red and green channels within the same field is shown (merge). The relative interaction percent was calculated by Image J. (scale bar, 10 μm).

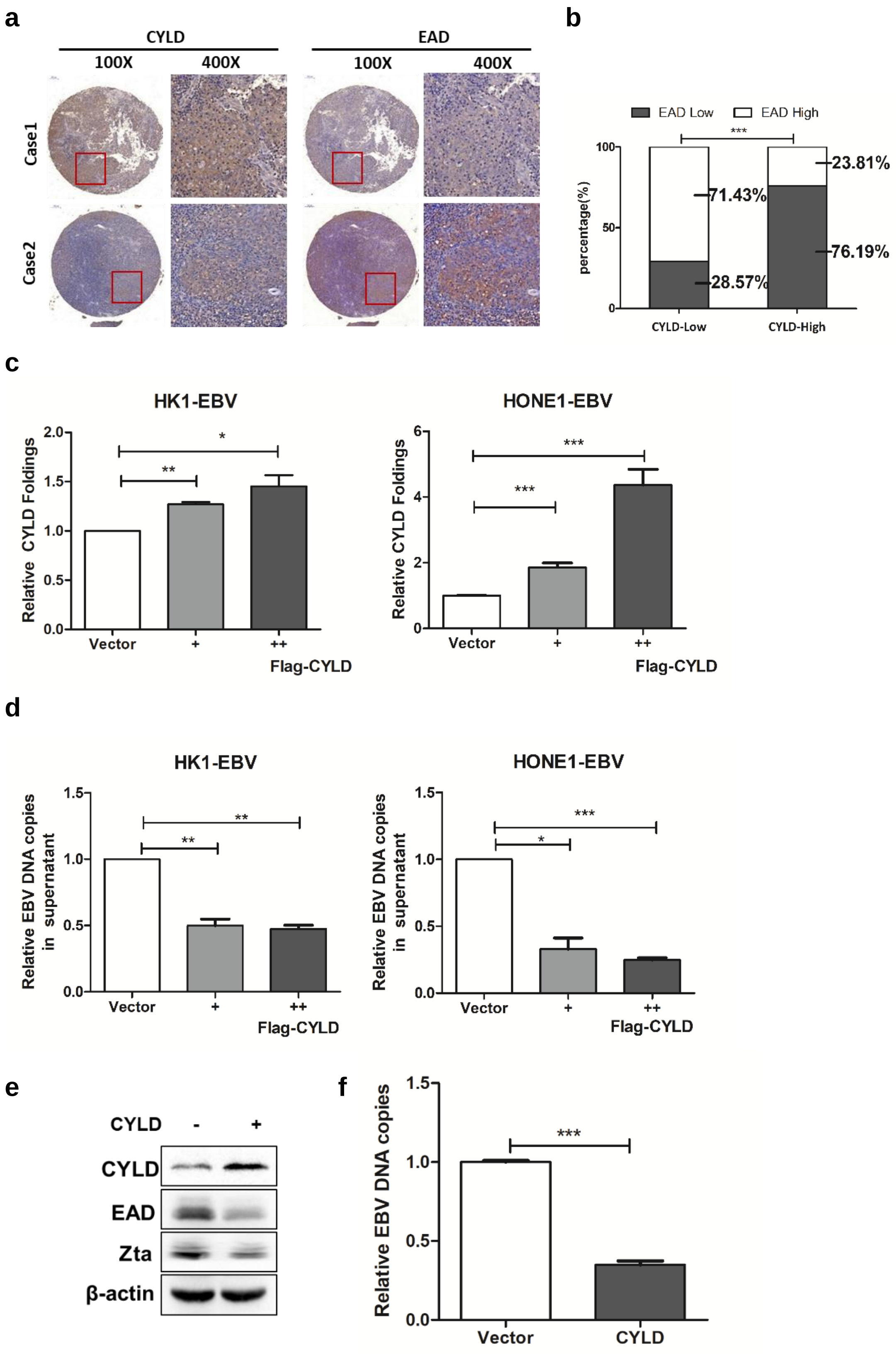

Supplementary Figure 9. CYLD is negatively related with EBV reactivation

a Representative IHC staining of CYLD and EAD expression from a NPC tissue

microarray (100×: scale bar, 100 μm; 400×: scale bar, 50 μm). **b** EAD expression level was calculated based on CYLD expression. C-D) HK1-EBV and HONE1-EBV cells were infected with vector or CYLD. **c** Total RNA from cells was isolated and subjected to real-time PCR. **d** Real-time PCR showing EBV DNA copy levels of EBV in HK1-EBV and HONE1-EBV cell culture medium supernatant fractions. **e** HONE1-EBV cells implanted into nude mice were disrupted and analyzed by using Western blotting. **f** EBV DNA copies in tumors were analyzed by qPCR. (*$p < 0.05$, **$p < 0.01$, ***$p < 0.001$)

Supplementary Table 1. The clinical characteristics of 29 NPC patients and 20 NP patients

	Characteristics	Number of patients(%)
**NP**	**Gender**	
	Male	13（65）
	Female	7（35）
	**Age (years）**	
	＞51	7（35）
	≤51	13（65）
**NPC**	**Gender**	
	Male	22（75.86)
	Female	7（24.14）
	**Age (years）**	
	＞51	14（48.28）
	≤51	15（51.72）
	**WHO histological classification**	
	NKUC（UD）	26（89.66）
	NKUC（DF）	3（10.34）

Supplementary Table 2. Clinical characteristics and CYLD expression level in 44 non-keratinizing undifferentiated NPC patients.

Clinical Characteristics	CYLD Low expression	CYLD High expression	p-value
**Age (yr), mean ± S.D.**	44.18±9.53	41.68±11.22	0.43[a]
**Gender**			0.5[b]
**Males(n)**	17	18	
**Females(n)**	5	4	
**Neck lymph node metastasis**			
**Negative (n)**	12	8	0.316[b]
**Positive (n)**	10	11	
**No information (n)**	0	3	
**EBV infection**			
**Negative**	0	0	
**Positive**	22	22	
**Median survival time (monthz)**	48	72	

[a]Welch's t test.
[b]Pearson's χ^2 test.

Supplementary Table 3. Clinical characteristics and CYLD expression level in 129 NPC patients.

Clinical Characteristics	CYLD Low	CYLD High	p-value
Age(yr), mean±SD	48.13±11.47	45.47±11.48	0.128[a]
Gender			
Males(n)	57	58	0.542[b]
Females(n)	17	13	
Neck lymph node Metastasis			
Negative(n)	23	14	0.366[b]
Positive(n)	48	44	
Recurrence			
yes	37	22	0.021[b]
no	29	41	
EBV infection			
Negative(n)	1	1	
Positive(n)	70	57	

[a]Welch's t test.
[b]Pearson's χ^2 test.

Supplementary Table 4. Correlation between CYLD and EAD in 29 NPC patients

			EAD	
Biopsies	Spearman's rho	CYLD	Correlation coefficient	-0.46
			Significance (2-Tailed)	＜0.001
			N	29

Supplementary Table 5. Correlation between CYLD and EAD in a tissue microarray of NPC patients

			EAD	
Biopsies	Spearman's rho	CYLD	Correlation co-efficient	-0.40
			Significance (2-Tailed)	0.001
			N	44

Supplementary Table 6. Correlation between CYLD and p18 in a tissue microarray of NPC patients

			p18	
Biopsies	Spearman's rho	CYLD	Correlation coefficient	0.51
			Significance (2-Tailed)	＜0.001
			N	44

Supplementary Table 7. Primer sequences

Primer	Sequence
CYLD F	TGCCTTCCAACTCTCGTCTTG
CYLD R	AATCCGCTCTTCCCAGTAGG
β-actin F	CCAAGGCCAACCGCGAGAAGATGAC
β-actin R	AGGGTACATGGTGGTGCCGCC AGAC
BZLF1 F	CATGTTTCAACCGCTCCGACTGG
BZLF1 R	GCGCAGCCTGTCATTTTCAGATG
BMRF1 F	CTAGCCGTCCTGTCCAAGTGC
BMEF1 R	AGCCAAACGCTCCTTGCCCA
p18 F	CATCATGCTGCCTGGTTAGG
p18 R	GCTGGCCGTGTGCTTCACCA

Un-cropped Images of Blots

Figure 1

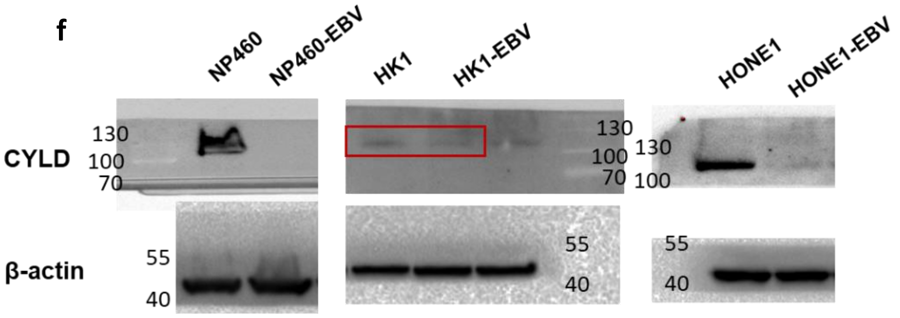

Figure 3

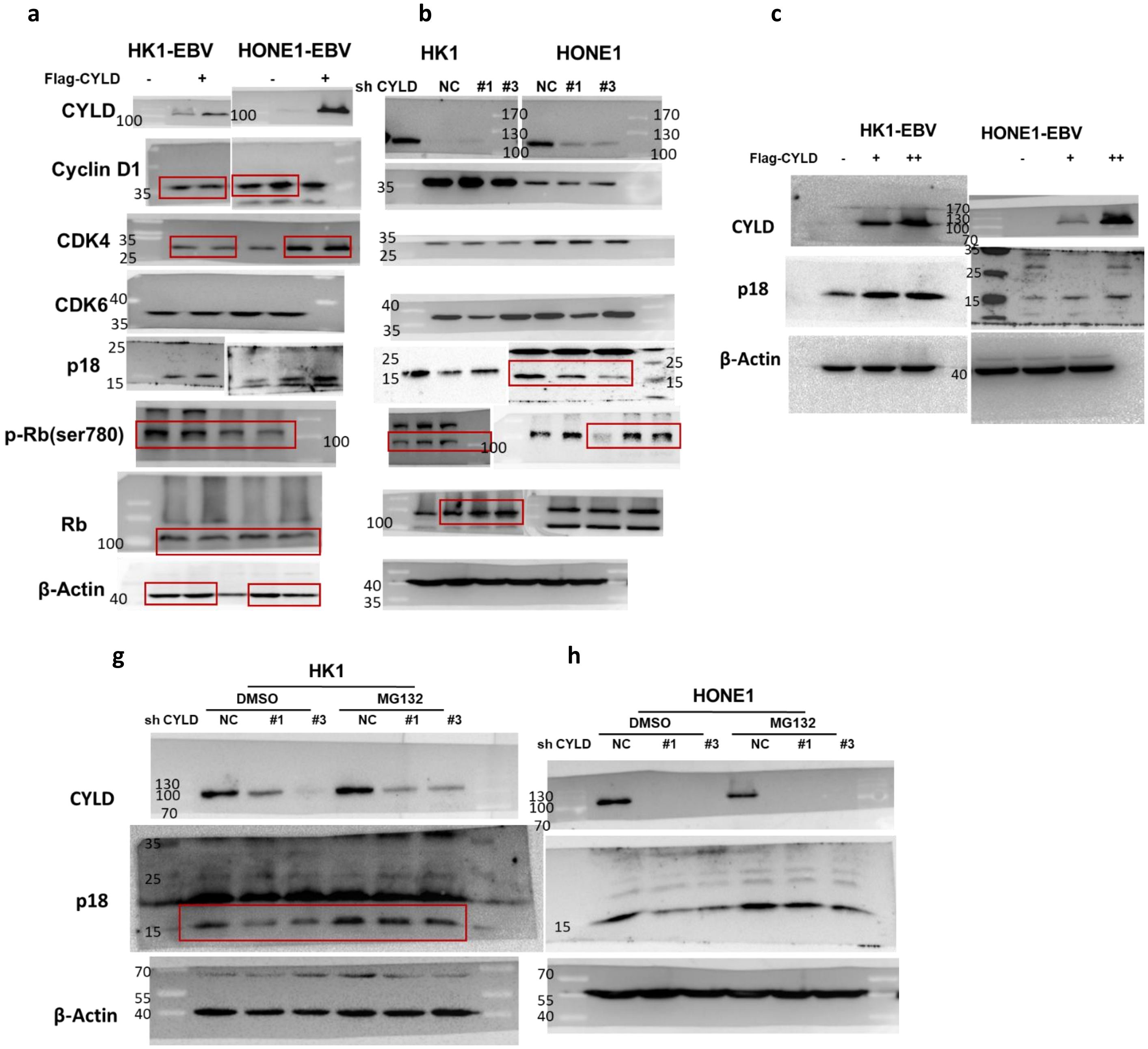

Figure 4

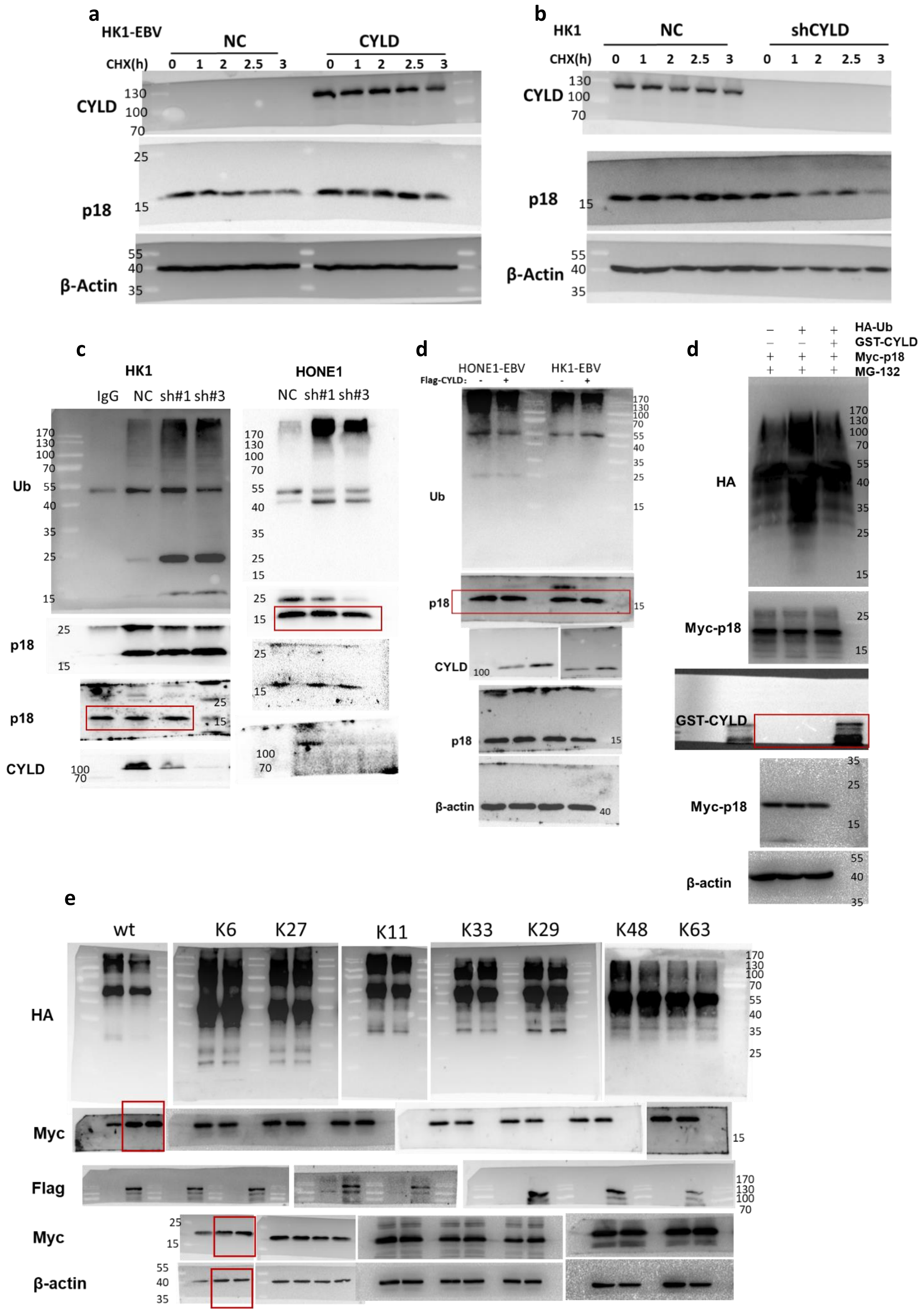

a
HK1-EBV
NC
CYLD
CHX(h) 0 1 2 2.5 3 0 1 2 2.5 3
CYLD
p18
β-Actin
b
HK1
NC
shCYLD
CHX(h) 0 1 2 2.5 3 0 1 2 2.5 3
CYLD
p18
β-Actin
c
HK1
IgG NC sh#1 sh#3
HONE1
NC sh#1 sh#3
Ub
p18
p18
CYLD
d
HONE1-EBV
HK1-EBV
Flag-CYLD:
Ub
p18
CYLD
p18
β-actin
d
HA-Ub
GST-CYLD
Myc-p18
MG-132
HA
Myc-p18
GST-CYLD
Myc-p18
β-actin
e
wt
K6
K27
K11
K33
K29
K48
K63
HA
Myc
Flag
Myc
β-actin

Figure 5

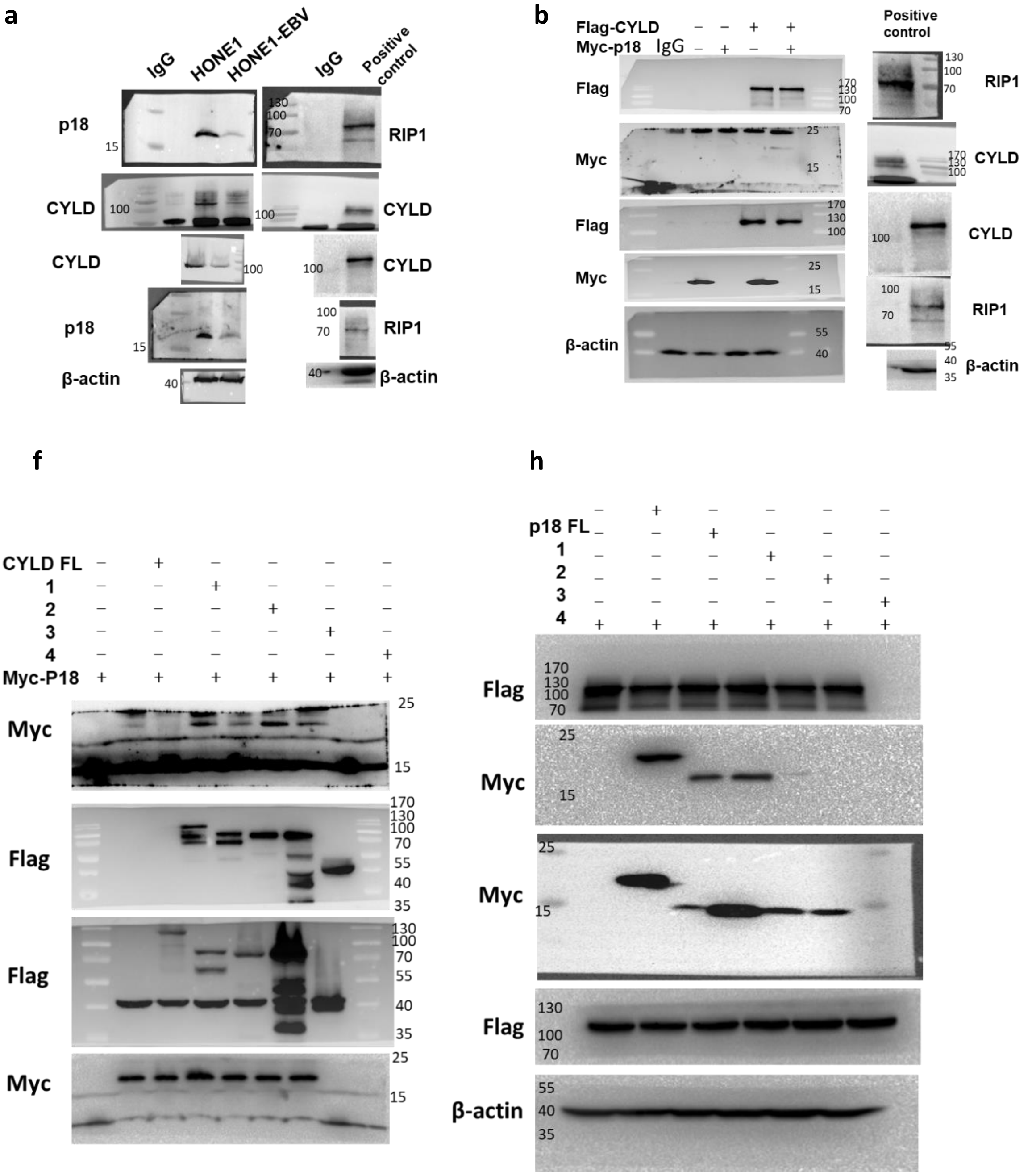

a
IgG
HONE1
HONE1-EBV
IgG
Positive control
p18
15
130
100
70
RIP1
CYLD
100
100
CYLD
CYLD
100
100
CYLD
p18
15
100
70
RIP1
β-actin
40
40
β-actin
b
Flag-CYLD
Myc-p18
IgG
Positive control
Flag
170
130
100
70
RIP1
Myc
25
15
CYLD
Flag
170
130
100
CYLD
Myc
25
15
100
70
RIP1
β-actin
55
40
35
β-actin
f
CYLD FL
1
2
3
4
Myc-P18
Myc
25
15
Flag
170
130
100
70
55
40
35
Flag
130
100
70
55
40
35
Myc
25
15
h
p18 FL
1
2
3
4
Flag
170
130
100
70
Myc
25
15
Myc
25
15
Flag
130
100
70
β-actin
55
40
35

Figure 6

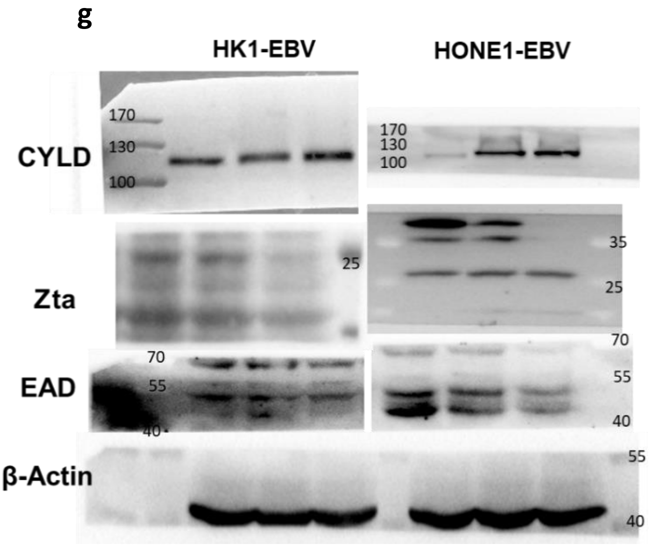

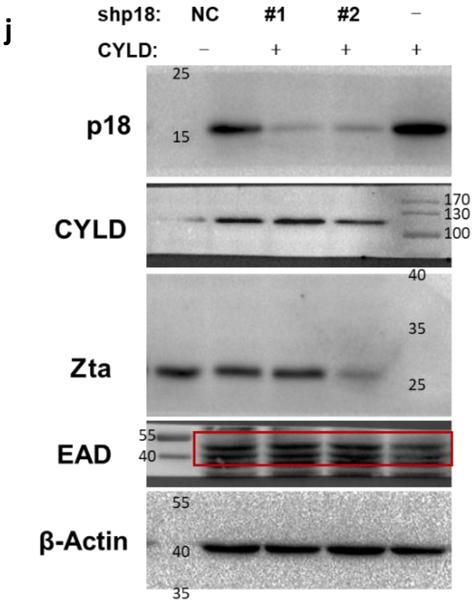

Figure 7

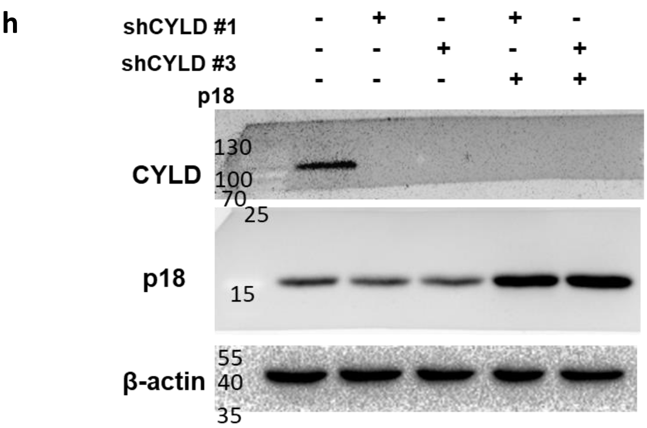

Supplementary Figure 6

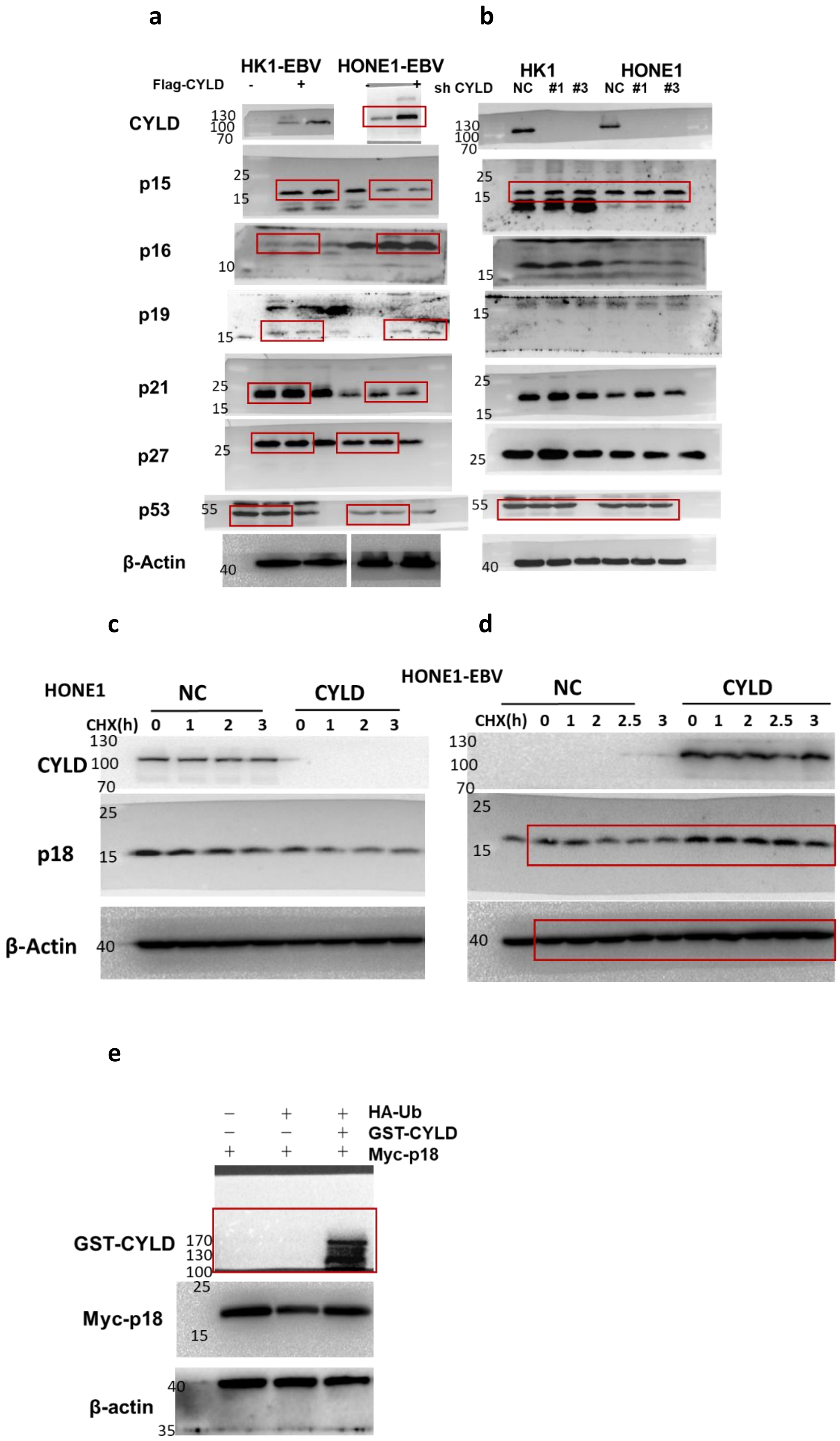

Supplementary Figure 9

e

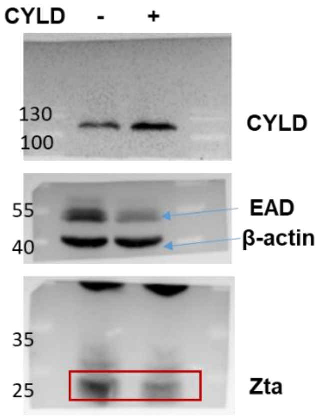

Supplement: Supplementary file 1 — Supplementary materials [file 41698_2021_153_MOESM1_ESM.pdf]
